# Total Syntheses and Stereochemical Assignment of Acremolides A and B

**DOI:** 10.3390/molecules29153599

**Published:** 2024-07-30

**Authors:** Yi Xiao, Junyang Liu, Yangyang Jiang, Yian Guo, Tao Ye

**Affiliations:** 1 State Key Laboratory of Chemical Oncogenomics, Peking University Shenzhen Graduate School, Shenzhen 518055, China; 2001112078@pku.edu.cn (Y.X.); liujy@wyu.edu.cn (J.L.); yangyangjiang@stu.pku.edu.cn (Y.J.); 2 School of Pharmacy and Food Engineering, Wuyi University, Jiangmen 529020, China; 3 Qian Yan (Shenzhen) Pharmatech. Ltd., Shenzhen 518172, China

**Keywords:** total syntheses, natural products, asymmetric crotylation, acremolide

## Abstract

The absolute stereochemical configurations of acremolides A and B were predicted by a biochemistry-based rule and unambiguously confirmed through their total syntheses. The features of the total syntheses include sequential Krische’s Ir-catalyzed crotylation, Brown’s borane-mediated crotylation, Mitsunobu esterification reaction, and cross-metathesis reaction. The efficient total synthesis enabled clear validation of the predicted stereochemistry for acremolides A and B.

## 1. Introduction

Originally identified by Capon et al. in 2007, acremolides A–D were isolated from *Acremonium* sp. (MST-MF588a) discovered in an Australian marine-derived fungus [1]. Acremolides A–D represent a novel class of lipodepsipeptides characterized by a 12-membered macrocyclic lactam that is composed of a dipeptide unit and a substituted fatty acid fragment. Utilizing a novel C_3_ Marfey’s method [2], Capon and co-workers successfully determined the absolute configurations of the amino acid fragments within the molecule, namely, L-Pro and D-Phe. However, due to the unsuccessful coupling of the acremolides A and B with (*S*)-Mosher’s reagent [3], the relative and absolute stereochemistry of the fatty acid within these natural products remains unassigned. Confirming the absolute stereochemistry of natural products through total synthesis is a well-established approach [4,5,6,7,8]. Prior efforts have not yet unveiled the stereochemistry of acremolides. In 2010, Cossy’s group accomplished the total synthesis of the one and only stereoisomer of acremolide B. Putatively, the synthesis of the remaining 15 stereoisomers is necessary to determine its absolute stereochemistry [9]. In 2017, Pabbaraja’s group endeavored to synthesize multiple isomers of acremolide B to elucidate its absolute configuration but encountered difficulties in macrocyclization via esterification [10]. Thus, the absolute stereochemistry of acremolides A–D remains elusive. For many years, our research has focused on elucidating the absolute stereochemistry of cyclic peptide natural products through total synthesis [11,12]. This work enables further investigations into the structure–activity relationships (SARs) of these biologically active natural products. We are interested in the absolute configurations of acremolides A and B, and to this end, we embarked on the synthesis of these two natural products aimed toward the assignment of their stereochemical structures.

**Figure 1:**
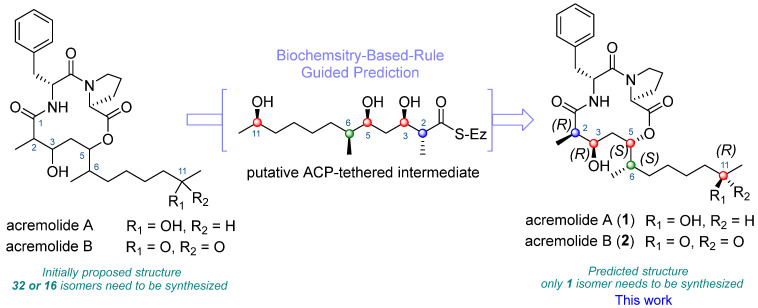
Biochemistry-based-rule guided prediction of the structure of acremolides A and B.

Fungal HR-PKSs (highly reducing polyketide synthases) are single-module enzymes that catalyze the elongation of linear fatty acyl chains with exquisite stereochemical control. Recently, we and co-workers discovered a unified stereochemical course for polyhydroxy PKs, phialotides, phomenoic acid, and ACR-toxin (acyl carrier protein toxin), and we theorized the biochemistry-based rule for the configuration prediction of fungal-reduced polyketides [13]. Considerations on the biosynthesis of acremolides A and B and the rules related to the prediction of stereochemical configuration (please see the Supplementary Materials for details) led to the proposal that its previously unknown stereostructure should be represented as illustrated in Figure 1. Thus, the ketoreductase (KR) domain reduces the corresponding keto group to give the *R*-configured C11, *S*-configured C5, and *R*-configured C3 (marked in red); the enoyl reductase domain iteratively reduces enoyl moieties to give the *S*-configured C6 (marked in green); and the methyltransferase (MT) domain installs a methyl group at the α position to give the *R*-configured C2 (marked in blue). As such, we predict the absolute configuration of the acremolides A and B as depicted in Figure 1. The following task is to synthesize these compounds to substantiate our hypothesis.

**Scheme 1:**
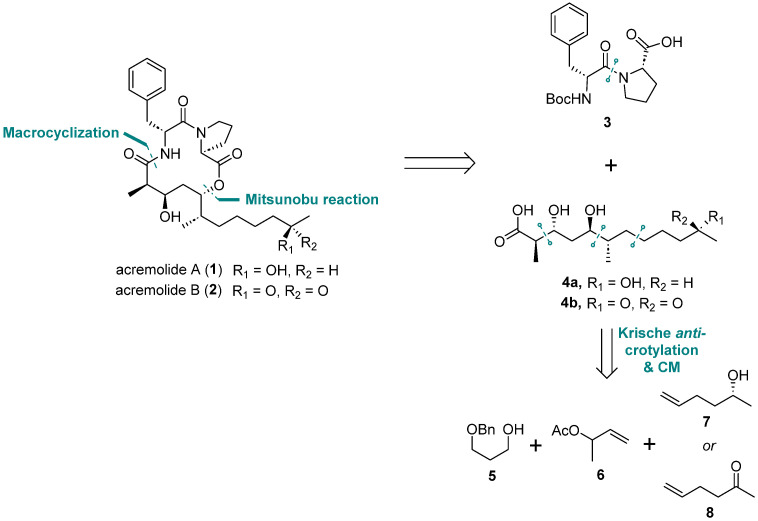
Retrosynthetic analysis of acremolides A (**1**) and B (**2**).

As shown in Scheme 1, our retrosynthetic analysis indicates that acremolides A and B could be prepared from dipeptide **3** and fatty acid **4** via a Mitsunobu reaction [14] and macrocyclization to form the 12-membered ring. It was further envisioned that intermediate **4** could be produced by a double Krische’s asymmetric *anti*-crotylation [15] and an olefin cross-metathesis reaction [16,17], tracing back to four key compounds: **5**, **6**, **7**, and **8**. Compared to acremolide A, acremolide B has one less stereogenic center and requires fewer synthetic steps. Therefore, our initial endeavor was focused on the access to acremolide B.

## 2. Results and Discussion

**Scheme 2:**
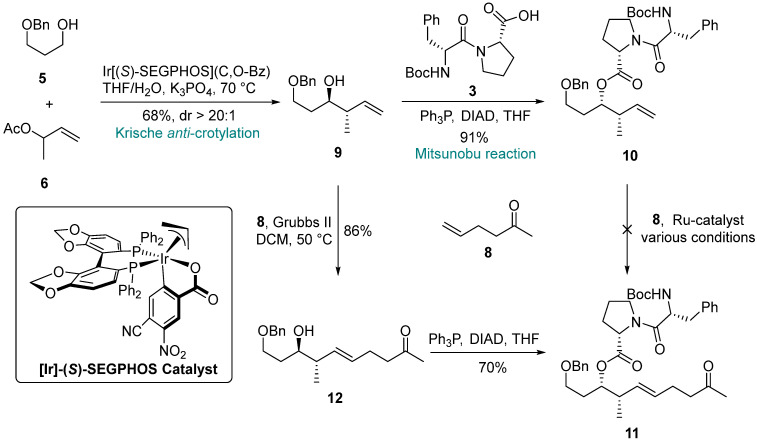
Synthesis of ester **11**.

Our synthesis of acremolide B commenced with the conversion of the known compounds **5** and **6** into homoallylic alcohol **9** (Scheme 2). Utilizing Krische’s methodology [15] with the chiral iridium catalyst Ir(S)-SEGPHOS, potassium phosphate, and heating at 70 °C in THF, we converted these compounds to product **9** with a remarkable yield of 68%, accompanied by an impressive stereoselectivity of dr > 20:1. Compound **9** was then esterified with dipeptide fragment **3** under Mitsunobu conditions (Ph_3_P, DIAD), yielding ester **10** in a high yield of 91%. Our efforts then focused on introducing the fatty acid side chain to obtain the crucial intermediate **11** through an olefin cross-metathesis reaction involving terminal olefins **10** and **8**. Extensive conditions were surveyed, which included screening various catalysts such as the Hoveyda–Grubbs II [18,19] and Grubbs II catalysts [20,21], along with exploring different solvents and temperatures. However, these two fragments failed to undergo intermolecular olefin cross-metathesis reaction. This observed behavior can arguably be attributed to the spatial hindrance present at the olefinic α and β positions of olefin **10**, which was considered as a type IV olefin [22]. Alternatively, less hindered olefin **9** underwent cross-metathesis smoothly with olefin **8** in a heated (50 °C) sealed tube in the presence of Grubbs II catalyst, resulting in an 86% yield of product **12**. Subsequently, intermediate **12** was subjected to a Mitsunobu reaction to furnish the key fragment **11** with a moderate yield of 70%.

**Scheme 3:**
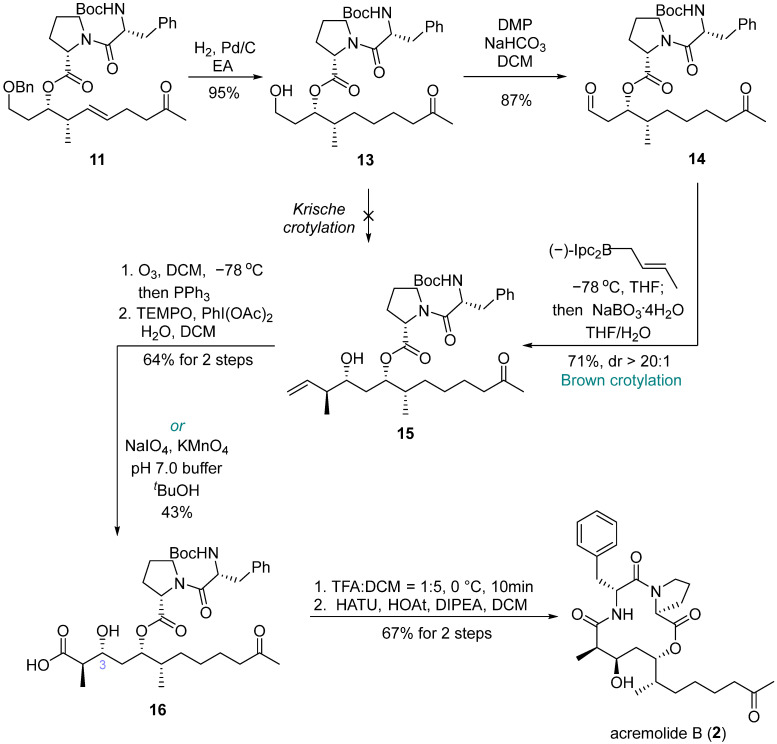
Total synthesis of acremolide B (**2**).

With compound **11** in hand, our phase was set for the total synthesis of acremolide B (Scheme 3). Hydrogenation of olefin **11** using 10% Pd/C under a hydrogen atmosphere afforded compound **13** with 95% high yield. We opted for Brown crotylation reaction [23,24] to achieve this transformation. Initially, alcohol **13** was converted to aldehyde **14** via Dess–Martin periodinane oxidation [25]. The resulting aldehyde was then subjected to standard Brown crotylation conditions, affording alcohol **15** in a moderate yield of 71%, with a high stereoselectivity of dr > 20:1. Under Krische’s conditions, oxidative cleavage of the terminal double bond to carboxylic acid was achieved in a single step using NaIO_4_ and KMnO_4_ [26] as co-oxidants in a pH = 7.0 buffered solvent, albeit with a modest yield of 43%. To improve the yield of this transformation, a two-step process was implemented. It involved ozonolysis to cleave the terminal double bond to an aldehyde, followed by a Pinnick oxidation reaction to obtain carboxylic acid **16**. However, we found that the secondary hydroxyl group at C3 underwent an elimination reaction under the Pinnick oxidation conditions. To prevent the elimination reaction, we chose the milder TEMPO/PhI(OAc)_2_ oxidation system [27], ultimately affording carboxylic acid **16** in two steps with an overall yield of 64%.

Recognizing the potential risk of C3 secondary hydroxyl group elimination under acidic Boc- removal conditions, we optimized the reaction condition. Ultimately, we found that the Boc- group was successfully removed in 10 min by employing a TFA/DCM solvent with a volume ratio of 1:5 at 0 °C, and the occurrence of the elimination reaction was also prevented effectively. Finally, the macrocyclization was performed under HATU/HOAt/DIPEA conditions, affording acremolide B (**2**) in two steps with an overall yield of 67%. The ^1^H and ^13^C NMR spectra data and the optical rotation for our synthetic acremolide B (**2**) were consistent with the literature values. As such, we assigned the structure of acremolide B (**2**) as depicted in Figure 1.

**Scheme 4:**
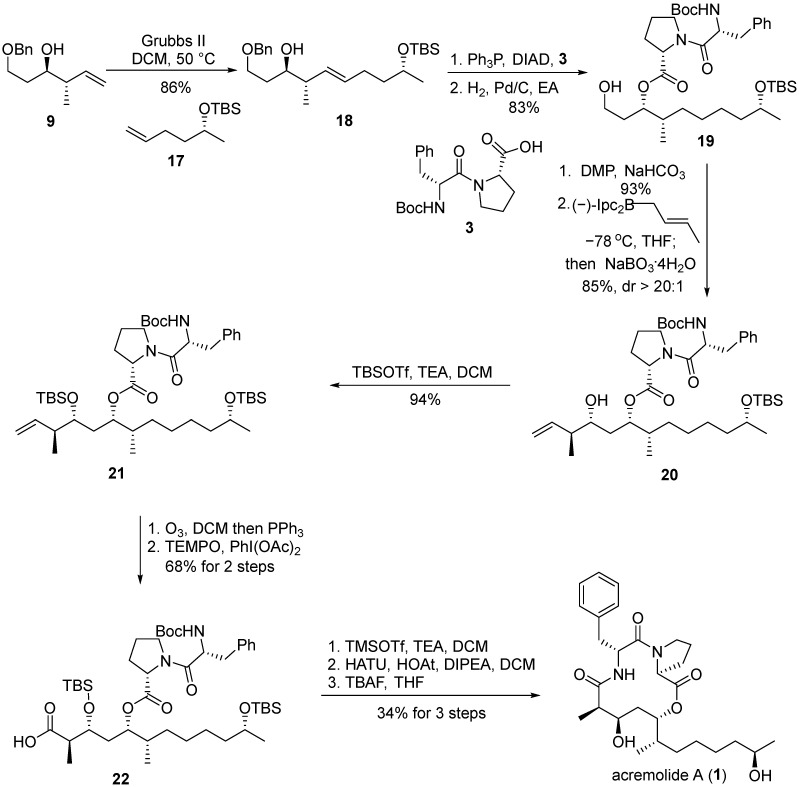
Total synthesis of acremolide A (**1**).

Upon completing the total synthesis of acremolide B and establishing its absolute stereochemistry, our next task is to explore the total synthesis of acremolide A and unveil its stereochemistry. Given the structural similarity between acremolide A and B, the synthetic route to acremolide B was similarly applied to acremolide A, as shown in Scheme 4.

The cross-metathesis of terminal olefins **9** and **17** with Grubbs II catalyst generated compound **18** in a good yield of 86%. Mitsunobu esterification of alcohol **18** and carboxylic acid **3** followed by hydrogenation of the double bond and simultaneous deprotection of the Bn- protecting group yielded **19** in an overall yield of 83% over two steps. Subsequently, Dess–Martin periodinane oxidation of **19** afforded an aldehyde (not shown), which was in turn subjected to Brown crotylation conditions, furnishing intermediate **20** in a good yield of 85%, with a remarkable stereoselectivity of dr > 20:1. Protection of the secondary hydroxyl group in **20** with TBS silyl group furnished **21** without incident. Following this, cleavage of the double bond by ozone and subsequent oxidation of the resulting aldehyde to a carboxylic acid under TEMPO/PhI(OAc)_2_ conditions resulted in compound **22** in 68% overall yield. Eventually, deprotection of the Boc- protection group under TMSOTf/TEA conditions, followed by macrocyclization using HATU/HOAT/DIPEA conditions, and subsequent removal of the TBS silyl group, resulted in the synthesis of natural product acremolide A in 34% overall yield in three steps (**1**). The ^1^H and ^13^C NMR spectra data and the optical rotation for our synthetic acremolide A (**1**) were consistent with the literature values. On theses basis, we assigned the structure of acremolide A (**1**) as depicted in Figure 1.

## 3. Materials and Methods

### 3.1. General Experimental Details

All reactions were conducted in flame-dried or oven-dried glassware under an atmosphere of dry nitrogen or argon. Oxygen and/or moisture-sensitive solids and liquids were transferred appropriately. The concentration of solutions in vacuo was accomplished using a rotary evaporator fitted with a water aspirator. Residual solvents were removed under a high vacuum (0.1–0.2 mm Hg). All reaction solvents were purified before use: Tetrahydrofuran (THF) was distilled from Na/benzophenone. Toluene was distilled over molten sodium metal. Dichloromethane (DCM), 1,2-dichloroethane (DCE) and trimethylamine (Et_3_N) were distilled from CaH_2_. Methanol (MeOH) was distilled from Mg/I_2_. The reagents were purchased at the highest commercial quality and used without further purification unless otherwise stated. Flash column chromatography was performed using the indicated solvents on silica gel 60 (230–400 mesh ASTM E, Qingdao, China). Reactions were monitored using thin-layer chromatography (TLC), which was carried out using pre-coated sheets (Qingdao silica gel 60-F250, 0.2 mm). Compounds were visualized with UV light, iodine, and ceric ammonium molybdate stainer phosphomolybdic acid in EtOH. The ^1^H NMR spectra were recorded on Avance 400 MHz spectrometers (Bruker, Karlsruhe, Germany). Chemical shifts were reported in parts per million (ppm), relative to either a tetramethylsilane (TMS) internal standard or the signals due to the solvent. The following abbreviations are used to describe the spin multiplicity: s = singlet; d = doublet; t = triplet; q = quartet; qn = quintet; m = multiplet; br = broad; dd = doublet of doublets; dt = doublet of triplets; dq = doublet of quartets; ddd = doublet of doublet of doublets. Other combinations are derived from those listed above. Coupling constants (J) are reported in Hertz (Hz) for corresponding solutions, and chemical shifts are reported as parts per million (ppm) relative to residual CHCl_3_ δH (7.26 ppm). ^13^C-NMR nuclear magnetic resonance spectra were recorded at 100 MHz, 125 MHz, or 150 MHz for corresponding solutions, and chemical shifts are reported as parts per million (ppm) relative to residual CDCl_3_ δC (77.16 ppm). High-resolution mass spectra were measured on an ABI Q-star Elite (Applied Biosystems, Beijing, China). Optical rotations were recorded on a Rudolph AutoPol-I polarimeter (Shanghai, China) at 589 nm with a 50 mm cell. Data are reported as follows: specific rotation (c (g/100 mL), solvent).

#### Synthesis of Compounds [28]

(3*R*,4*S*)-1-(benzyloxy)-4-methylhex-5-en-3-ol **9**:

A pressure tube was charged with alcohol **5** (43 mg, 0.26 mmol, 1.0 equiv.), catalyst [Ir] (14 mg, 0.013 mmol, 0.05 equiv.), alkene **6** (89 mg, 0.78 mmol, 3.0 equiv.), and potassium phosphate (62 mg, 0.29 mmol, 1.1 equiv.). The pressure tube was carefully purged with argon and degassed tetrahydrofuran (0.26 mL, 1.0 M) was added, followed by the addition of distilled water (24 µL, 1.3 mmol, 5.0 equiv.). The tube was sealed, avoiding air contamination, and then heated to 70 °C for 48 h in an oil bath. The reaction mixture was allowed to cool to room temperature and then diluted with dichloromethane (10 mL). Solids were filtered off using a celite pad and rinsed with dichloromethane (3 × 5 mL). Solvents were evaporated to give a brown oil. The crude material was absorbed on silica gel and purified by flash chromatography (ethyl acetate/hexanes = 1/9) to afford alcohol **9** (39 mg, 68%) as a colorless oil. TLC: R*_f_* = 0.30 (silica gel, ethyl acetate/hexanes = 1/9). UV and PMA stain. ${\left[\alpha \right]}_{D}^{26}$ = −4.3 (*c* 1.0, CH_3_OH). ^1^H NMR (400 MHz, CDCl_3_) δ 7.54–7.17 (m, 5H), 6.10–5.70 (m, 1H), 5.13 (s, 1H), 5.10 (d, *J* = 8.8 Hz, 1H), 4.56 (s, 2H), 3.83–3.63 (m, 3H), 2.82 (s, 1H), 2.89–2.74 (m, 1H), 1.78 (q, *J* = 7.8, 6.8 Hz, 2H), 1.08 (d, *J* = 6.9 Hz, 3H). ^13^C NMR (101 MHz, CDCl_3_) δ 140.6, 138.0, 128.5, 127.8, 115.6, 74.3, 73.4, 69.3, 44.1, 33.6, 15.9. HRMS (ESI, *m*/*z*) for C_14_H_21_O_2_^+^ [M+H]^+^: Calcd. 221.1536; found: 221.1537.

(3*S*,4*S*)-1-(benzyloxy)-4-methylhex-5-en-3-yl(*tert*-butoxycarbonyl)-*D*-phenylalanyl-*L*-prolinate **10**:

To a solution of alcohol **9** (34 mg, 0.16 mmol, 1.0 equiv.) and acid **3** (112 mg, 0.31 mmol, 2.0 equiv.) in anhydrous THF (2.0 mL, 0.08 M) at 0 °C under an argon atmosphere, PPh_3_ (122 mg, 0.47 mmol, 3.0 equiv.) and DIAD (61 µL, 0.31 mmol, 2.0 equiv.) were added. After being stirred at room temperature for 4 h, the reaction mixture was quenched with saturated aqueous solution of NaHCO_3_ (10 mL) and extracted with EtOAc (3 × 10 mL). The combined organic layers were washed with saturated aqueous solution of brine (20 mL), dried over anhydrous Na_2_SO_4_, filtered, and concentrated in vacuo. Purification of the crude product was performed by flash chromatography on silica gel (hexanes/EtOAc = 4/1) to afford **10** (80 mg, 91%) as a colorless oil. TLC: R*_f_* = 0.40 (silica gel, ethyl acetate/hexanes = 1/4). UV and PMA stain. ${\left[\alpha \right]}_{D}^{26}$ = −48.60 (*c* 1.0, CH_3_OH). ^1^H NMR (400 MHz, CDCl_3_) δ 7.39–7.32 (m, 4H), 7.32–7.20 (m, 6H), 5.75 (ddd, *J* = 17.5, 9.8, 7.2 Hz, 1H), 5.41 (d, *J* = 8.6 Hz, 1H), 5.14–5.05 (m, 2H), 5.03 (d, *J* = 4.9 Hz, 1H), 4.66 (td, *J* = 9.0, 5.4 Hz, 1H), 4.53 (q, 2H), 4.33 (dd, *J* = 8.2, 3.7 Hz, 1H), 3.64–3.44 (m, 3H), 3.08 (dd, *J* = 12.8, 5.5 Hz, 1H), 2.95 (dd, *J* = 12.7, 9.4 Hz, 1H), 2.61 (dt, *J* = 9.5, 6.8 Hz, 1H), 2.43 (q, *J* = 6.7 Hz, 1H), 2.00–1.73 (m, 5H), 1.43 (s, 9H), 1.37–1.32 (m, 1H), 1.02 (d, *J* = 6.8 Hz, 3H). ^13^C NMR (101 MHz, CDCl_3_) δ 171.5, 170.1, 155.0, 139.7, 138.5, 136.6, 129.6, 128.4, 128.4, 128.0, 127.6, 127.0, 115.4, 79.7, 74.7, 73.3, 66.8, 59.1, 53.6, 46.8, 41.5, 40.5, 31.9, 29.1, 28.4, 24.4, 21.8, 14.8. HRMS (ESI, *m/z*) for C_33_H_45_N_2_O_6_^+^ [M+H]^+^: Calcd. 565.3272; found: 565.3270.

(7*S*,8*R*,*E*)-10-(benzyloxy)-8-hydroxy-7-methyldec-5-en-2-one **12**:

To a solution of **9** (16 mg, 0.073 mmol, 1.0 equiv.) and alkene **8** (14 mg, 0.15 mmol, 2.0 equiv.) in DCM (1.0 mL, 0.07 M) at room temperature, Grubbs II catalyst (13 mg, 0.015 mmol, 0.2 equiv.) was added. The reaction mixture was heated at 50 °C for 9 h before it was cooled back to room temperature and concentrated in vacuo. Purification of the crude product was performed by flash chromatography on silica (hexanes/EtOAc = 4/1) to afford **12** (18 mg, 86%) as a colorless oil. TLC: R*_f_* = 0.20 (silica gel, ethyl acetate/hexanes = 1/4). UV and PMA stain. ${\left[\alpha \right]}_{D}^{28}$ = −13.20 (*c* 1.0, CH_3_OH). ^1^H NMR (400 MHz, CDCl_3_) δ 7.45–7.30 (m, 5H), 5.61–5.32 (m, 2H), 4.56 (s, 2H), 3.88–3.50 (m, 3H), 2.76 (s, 1H), 2.54 (t, *J* = 7.3 Hz, 2H), 2.42–2.27 (m, 2H), 2.19 (d, *J* = 6.3 Hz, 1H), 2.17 (s, 3H), 1.82–1.70 (m, 2H), 1.04 (d, *J* = 6.9 Hz, 3H). ^13^C NMR (101 MHz, CDCl_3_) δ 208.6, 138.1, 133.0, 129.8, 128.5, 127.8, 74.4, 73.4, 69.3, 43.4, 43.1, 33.8, 31.5, 30.1, 27.0, 16.6. HRMS (ESI, *m*/*z*) for C_18_H_27_O_3_^+^ [M+H]^+^: Calcd. 291.1955; found: 291.1955.

(3*S*,4*S*,*E*)-1-(benzyloxy)-4-methyl-9-oxodec-5-en-3-yl(*tert*-butoxycarbonyl)-*D*-phenylalanyl-*L*-prolinate **11**:

To a solution of alcohol **12** (90 mg, 0.31 mmol, 1.0 equiv.) and acid **3** (230 mg, 0.63 mmol, 2.0 equiv.) in anhydrous THF (4.0 mL, 0.08 M) at 0 °C under an argon atmosphere, PPh_3_ (246 mg, 0.94 mmol, 3.0 equiv.) and DIAD (130 µL, 0.62 mmol, 2.0 equiv.) were added. After being stirred at room temperature for 9 h, the reaction mixture was quenched with saturated aqueous solution of NaHCO_3_ (10 mL) and extracted with EtOAc (3 × 10 mL). The combined organic layers were washed with saturated aqueous solution of brine (20 mL), dried over anhydrous Na_2_SO_4_, filtered, and concentrated in vacuo. Purification of the crude product was performed by flash chromatography on silica gel (hexanes/EtOAc = 3/1) to afford **11** (139 mg, 70%) as a colorless oil. TLC: R*_f_* = 0.40 (silica gel, ethyl acetate/hexanes = 1/3). UV and PMA stain. ${\left[\alpha \right]}_{D}^{26.9}$ = −38.80 (*c* 1.0, CHCl_3_). ^1^H NMR (400 MHz, CDCl_3_) δ 7.39–7.36 (m, 4H), 7.28–7.22 (m, 6H), 5.50–5.30 (m, 2H), 5.04–4.96 (m, 1H), 4.70–4.61 (m, 1H), 4.61–4.44 (m, 2H), 4.33 (dd, *J* = 8.2, 3.5 Hz, 1H), 3.62–3.47 (m, 3H), 3.09 (dd, *J* = 12.8, 5.4 Hz, 1H), 2.95 (dd, *J* = 12.8, 9.4 Hz, 1H), 2.66–2.57 (m, 1H), 2.55–2.47 (m, 2H), 2.35 (q, *J* = 6.8 Hz, 1H), 2.31–2.23 (m, 2H), 2.16 (s, 3H), 1.98–1.87 (m, 2H), 1.87–1.74 (m, 3H), 1.43 (s, 9H), 0.97 (d, *J* = 6.8 Hz, 3H). ^13^C NMR (101 MHz, CDCl_3_) δ 208.4, 171.6, 170.1, 155.0, 138.6, 136.6, 132.4, 129.6, 129.5, 128.4, 128.4, 128.0, 127.6, 127.0, 79.7, 75.1, 73.3, 66.8, 59.0, 53.6, 46.8, 43.3, 40.7, 40.5, 32.2, 30.0, 29.1, 28.4, 26.7, 24.4, 15.7. HRMS (ESI, *m*/*z*) for C_37_H_51_N_2_O_7_^+^ [M+H]^+^: Calcd. 635.3691; found: 635.3694.

(3*S*,4*S*)-1-hydroxy-4-methyl-9-oxodecan-3-yl(*tert*-butoxycarbonyl)-*D*-phenylalanyl-*L*-prolinate **13**:

To a solution of **11** (437 mg, 0.69 mmol, 1.0 equiv.) in EtOAc (15 mL, 0.05 M), palladium on charcoal (200 mg, 10% Pd, 0.19 mmol, 0.28 equiv.) was added under argon atmosphere. The reaction flask was evacuated and purged with hydrogen three times. The reaction mixture was stirred under a hydrogen atmosphere at room temperature for 9 h; the flask was then evacuated and purged with nitrogen three times, and the catalyst was removed by filtration through a pad of celite. The filtrate was concentrated under reduced pressure to afford crude product, which was purified by flash chromatography on silica gel (hexanes/EtOAc = 1/1) to afford **13** (358 mg, 95%) as a colorless oil. TLC: R*_f_* = 0.50 (silica gel, ethyl acetate/hexanes = 1/1). UV and PMA stain. ${\left[\alpha \right]}_{D}^{20.7}$ = −45.80 (*c* 1.0, CHCl_3_). ^1^H NMR (400 MHz, CDCl_3_) δ 7.22–7.09 (m, 5H), 5.41 (d, *J* = 8.6 Hz, 1H), 5.04–4.92 (m, 1H), 4.60–4.50 (m, 1H), 4.17 (dd, *J* = 8.4, 4.1 Hz, 1H), 3.67–3.55 (m, 2H), 3.49–3.38 (m, 1H), 2.98 (dd, *J* = 12.8, 5.4 Hz, 1H), 2.84 (dd, *J* = 12.8, 9.5 Hz, 1H), 2.61–2.49 (m, 1H), 2.34 (t, *J* = 7.3 Hz, 2H), 2.05 (s, 3H), 1.92–1.83 (m, 1H), 1.81–1.73 (m, 2H), 1.72–1.65 (m, 2H), 1.49–1.40 (m, 3H), 1.36 (s, 9H), 1.28–1.16 (m, 4H), 1.10–0.99 (m, 1H), 0.81 (d, *J* = 6.8 Hz, 3H). ^13^C NMR (101 MHz, CDCl_3_) δ 209.2, 172.6, 170.4, 155.0, 136.4, 129.5, 128.4, 127.0, 79.7, 75.1, 59.1, 58.6, 53.6, 46.9, 43.6, 40.3, 36.8, 34.7, 32.8, 29.9, 29.1, 28.4, 26.6, 24.6, 23.8, 14.5. HRMS (ESI, *m*/*z*) for C_30_H_47_N_2_O_7_^+^ [M+H]^+^: Calcd. 547.3378; found: 547.3377.

(3*S*,4*R*,6*S*,7*S*)-4-hydroxy-3,7-dimethyl-12-oxotridec-1-en-6-yl(*tert*-butoxycarbonyl)-*D*-phenylalanyl-*L*-prolinate **15**:

To a solution of alcohol **13** (237 mg, 0.43 mmol, 1.0 equiv.) in dry DCM (10 mL, 0.043 M) at 0 °C under argon atmosphere, Dess–Martin periodinane (221 mg, 0.52 mmol, 1.2 equiv.) was added. After being stirred at room temperature for 2 h, the reaction mixture was quenched with saturated aqueous solution of Na_2_SO_3_ (10 mL) and extracted with EtOAc (3 × 10 mL). The combined organic extracts were washed with saturated aqueous solution of copper sulfate (10 mL) and brine (10 mL), dried over anhydrous Na_2_SO_4_, filtered, and concentrated in vacuo. The residue was used directly in the next step without further purification.

To a suspension of *t*BuOK (246 mg, 2.2 mmol, 5.0 equiv.) in anhydrous THF (10 mL, 0.045 M) at −78 °C, *trans*-2-butene (172 mg, 3.1 mmol, 7.0 equiv.) was added, followed by the dropwise addition of *n*BuLi (1.4 mL, 2.2 mmol, 1.6 M in hexane, 5.0 equiv.). After the addition of *n*BuLi, the reaction mixture was allowed to warm to −45 °C and stirred for 30 min. After being re-cooled to −78 °C, a solution of (-)-Ipc_2_BOMe (840 mg, 2.6 mmol, 6.0 equiv.) in anhydrous THF (2 mL) was added and stirred for 30 min at −78 °C. BF_3_·Et_2_O (0.45 mL, 3.5 mmol, 8.0 equiv.) was then added, followed by a solution of aldehyde **14** (183 mg, 0.44 mmol, 1.0 equiv.) in THF (2 mL). After being stirred at −78 °C for 3 h, the reaction mixture was quenched with MeOH (5 mL) and allowed to warm to room temperature. Solvent was removed in vacuo and the residue was redissolved in THF (6 mL) and H_2_O (4 mL), and NaBO_3_·4H_2_O (339 mg, 2.2 mmol, 5.0 equiv.) was then added. The mixture was stirred at room temperature for 3 h and poured into a mixture of EtOAc (30 mL) and H_2_O (10 mL). The layers were separated, and the aqueous layer was further extracted with EtOAc (2 × 10 mL). The combined organic extracts were washed with brine (10 mL), dried over anhydrous Na_2_SO_4_, filtered, and concentrated in vacuo. Purification of the crude product was performed by flash chromatography on silica (hexanes/EtOAc = 2/1) to afford two separable diastereoisomers (187 mg, 71% total yield, dr > 20:1) with **21** (colorless oil) as the major product. TLC: R*_f_* = 0.40 (silica gel, ethyl acetate/hexanes = 1/2). UV and PMA stain. ${\left[\alpha \right]}_{D}^{23.3}$ = −45.80 (*c* 1.0, CHCl_3_). ^1^H NMR (400 MHz, CDCl_3_) δ 7.31–7.14 (m, 5H), 5.85 (ddd, *J* = 17.0, 10.8, 7.6 Hz, 1H), 5.45 (d, *J* = 8.5 Hz, 1H), 5.18–5.03 (m, 3H), 4.71–4.57 (m, 1H), 4.27 (dd, *J* = 8.2, 4.1 Hz, 1H), 3.70–3.62 (m, 1H), 3.55–3.47 (m, 1H), 3.09 (dd, *J* = 12.8, 5.4 Hz, 1H), 2.94 (dd, *J* = 12.8, 9.6 Hz, 1H), 2.61 (q, *J* = 7.6, 6.9 Hz, 1H), 2.45 (t, *J* = 7.3 Hz, 1H), 2.39–2.29 (m, 1H), 2.16 (s, 3H), 2.01–1.82 (m, 4H), 1.70–1.60 (m, 2H), 1.55 (t, *J* = 7.2 Hz, 1H), 1.47 (s, 9H), 1.38–1.26 (m, 6H), 1.10 (d, *J* = 6.9 Hz, 3H), 0.89 (d, *J* = 6.7 Hz, 3H). ^13^C NMR (101 MHz, CDCl_3_) δ 209.2, 172.0, 170.4, 155.1, 140.3, 136.6, 129.6, 128.5, 127.1, 115.6, 79.8, 72.9, 59.3, 53.8, 47.1, 44.3, 43.7, 40.6, 36.9, 36.4, 32.9, 30.0, 29.1, 28.5, 28.3, 26.8, 24.6, 23.9, 15.5, 14.3. HRMS (ESI, *m*/*z*) for C_34_H_53_N_2_O_7_^+^ [M+H]^+^: Calcd. 601.3847; found: 601.3849.

(2*R*,3*R*,5*S*,6*S*)-5-(((*tert*-butoxycarbonyl)-*D*-phenylalanyl-*L*-prolyl)oxy)-3-hydroxy-2,6-dimethyl-11-oxododecanoic acid **16**:

To a solution of alkene **15** (76 mg, 0.13 mmol, 1.0 equiv.) in DCM at −78 °C, ozone was bubbled through the solution until the solution became slightly blue. PPh_3_ (170 mg, 0.65 mmol, 5.0 equiv.) was added and the resultant solution was stirred at room temperature for 1 h. After concentration, the residue was used directly in the next step without further purification.

To a solution of the above crude aldehyde and PhI(OAc)_2_ (123 mg, 0.38 mmol, 3.0 equiv.) in DCM (10 mL, 0.013 M) at 0 °C, TEMPO (8 mg, 0.051 mmol, 0.4 equiv.) and H_2_O (1 mL) were added. The reaction mixture was warmed up to ambient temperature and stirred for 12 h, and then quenched with a saturated aqueous solution of Na_2_SO_3_ (10 mL). The aqueous layer was extracted with EtOAc (3 × 10 mL), and the combined organic layers were washed with H_2_O (10 mL) and brine (10 mL), dried over anhydrous Na_2_SO_4_, filtered, and concentrated in vacuo. Purification of the crude product was performed by flash chromatography on silica (hexanes/EtOAc = 1/1) to afford acid **16** (50 mg, 64%) as a colorless oil. TLC: R*_f_* = 0.10 (silica gel, ethyl acetate/hexanes = 1/1). UV and PMA stain. ${\left[\alpha \right]}_{D}^{26.3}$ = −31.20 (*c* 1.0, CHCl_3_). ^1^H NMR (400 MHz, CDCl_3_) δ 7.43–7.05 (m, 5H), 5.22–5.06 (m, 1H), 4.73–4.59 (m, 1H), 4.30–4.22 (m, 1H), 3.92–3.84 (m, 1H), 3.61–3.48 (m, 1H), 3.06 (dd, *J* = 12.8, 5.6 Hz, 1H), 2.99–2.86 (m, 1H), 2.72–2.57 (m, 2H), 2.48–2.37 (m, 2H), 2.14 (s, 3H), 2.00–1.77 (m, 5H), 1.59–1.49 (m, 4H), 1.45 (s, 9H), 1.37–1.19 (m, 8H), 0.88 (d, *J* = 6.7 Hz, 3H). ^13^C NMR (101 MHz, CDCl_3_) δ 209.4, 171.6, 155.2, 137.5, 136.4, 132.2, 132.1, 130.3, 129.5, 128.5, 127.5, 127.0, 79.9, 77.3, 59.3, 53.8, 47.2, 43.6, 40.2, 36.5, 32.8, 30.0, 29.7, 29.1, 28.4, 26.6, 24.5, 23.9, 14.2. HRMS (ESI, *m*/*z*) for C_33_H_51_N_2_O_9_^+^ [M+H]^+^: Calcd. 619.3589; found: 619.3585.

Acremolide B (**2**):

To a solution of **16** (36 mg, 0.06 mmol, 1.0 equiv.) in DCM (4 mL, 0.015 M) at 0 °C, TFA (0.8 mL) was added. The reaction mixture was allowed to be stirred for 10 min at 0 °C. The mixture was concentrated in vacuo directly. The residue was used directly in the next step without further purification.

To a solution of the above crude amine in DCM (60 mL, 0.001 M) at 0 °C, DIPEA (0.11 mL, 0.60 mmol, 10.0 equiv.), HATU (114 mg, 0.30 mmol, 5.0 equiv.), and HOAt (16 mg, 0.12 mmol, 2.0 equiv.) were added. The reaction mixture was allowed to warm for 24 h at room temperature and then concentrated in vacuo and the residue was redissolved in EtOAc (30 mL) and quenched with 4% aqueous citric acid solution. The aqueous layer was extracted with EtOAc (3 × 10 mL), and the combined organic layers were washed with saturated aqueous solution of NaHCO_3_ (10 mL) and brine (10 mL), dried over anhydrous Na_2_SO_4_, filtered, and concentrated in vacuo. Purification of the crude product was performed by flash chromatography on silica gel (hexanes/EtOAc = 1/2) to afford acremolide B (**2**) (20 mg, 67%) as a colorless oil. TLC: R*_f_* = 0.30 (silica gel, ethyl acetate/hexanes = 2/1). UV and PMA stain. ${\left[\alpha \right]}_{D}^{25}$ = −102 (*c* 0.02, MeOH). Major *cis* conformer: ^1^H NMR (400 MHz, DMSO-*d*_6_) δ 8.21 (d, *J* = 8.8 Hz, 1H), 7.38–7.12 (m, 5H), 5.00 (dd, *J* = 8.4, 3.2 Hz, 1H), 4.56–4.53 (m, 1H), 4.53–4.48 (m, 1H), 4.42 (d, *J* = 6.1 Hz, 1H), 3.78–3.69 (m, 1H), 3.57 (dd, *J* = 7.5, 5.0 Hz, 1H), 3.50–3.45 (m, 1H), 3.21 (dd, *J* = 13.9, 4.1 Hz, 1H), 2.83 (dd, *J* = 13.9, 10.8 Hz, 1H), 2.41 (t, *J* = 7.3 Hz, 2H), 2.33 (dd, *J* = 7.3, 2.1 Hz, 1H), 2.28–2.24 (m, 1H), 2.07 (d, *J* = 3.8 Hz, 3H), 1.97–1.88 (m, 2H), 1.83–1.79 (m, 1H), 1.78–1.75 (m, 1H), 1.74–1.71 (m, 1H), 1.70–1.66 (m, 1H), 1.44–1.42 (m, 1H), 1.38–1.37 (m, 1H), 1.22–1.13 (m, 1H), 1.17–1.14 (m, 1H), 0.96–0.89 (m, 1H), 0.83 (d, *J* = 6.8 Hz, 3H), 0.74 (d, *J* = 7.4 Hz, 3H). ^13^C NMR (101 MHz, DMSO-*d*_6_) δ 208.1, 175.6, 171.2, 168.9, 137.5, 128.8, 127.9, 127.7, 126.0, 75.1, 69.1, 57.7, 54.9, 48.4, 42.3, 41.7, 38.4, 35.1, 34.7, 31.2, 30.7, 29.3, 26.0, 25.4, 23.1, 20.3, 15.1, 15.0. (only for major isomer) HRMS (ESI, *m*/*z*) for C_28_H_40_N_2_O_6_Na^+^ [M+Na]^+^: Calcd. 523.2799; found 523.2797.

(3*R*,4*S*,9*R*,*E*)-1-(benzyloxy)-9-((*tert*-butyldimethylsilyl)oxy)-4-methyldec-5-en-3-ol **18**:

To a solution of **9** (100 mg, 0.46 mmol, 1.0 equiv.) and alkene **17** (195 mg, 0.91 mmol, 2.0 equiv.) in DCM (10 mL, 0.050 M) at room temperature, Grubbs II catalyst (77 mg, 0.091 mmol, 0.20 equiv.) was added. The reaction mixture was heated at 50 °C for 9 h before it was cooled back to room temperature and concentrated in vacuo. Purification of the crude product was performed by flash chromatography on silica (hexanes/EtOAc = 9/1) to afford **12** (159 mg, 86%) as a colorless oil. TLC: R*_f_* = 0.50 (silica gel, ethyl acetate/hexanes = 1/9). UV and PMA stain. ${\left[\alpha \right]}_{D}^{22}$ = −7.60 (*c* 3.0, CHCl_3_). ^1^H NMR (400 MHz, CDCl_3_) δ 7.43–7.31 (m, 5H), 5.59–5.44 (m, 1H), 5.43–5.32 (m, 1H), 4.56 (s, 2H), 3.87–3.79 (m, 1H), 3.79–3.72 (m, 1H), 3.72–3.60 (m, 2H), 2.90 (d, *J* = 3.4 Hz, 1H), 2.23 (q, *J* = 6.8 Hz, 1H), 2.17–2.07 (m, 1H), 2.06–1.98 (m, 1H), 1.90–1.79 (m, 1H), 1.78–1.65 (m, 1H), 1.61–1.40 (m, 2H), 1.16 (d, *J* = 6.0 Hz, 3H), 1.07 (d, *J* = 6.9 Hz, 3H), 0.93 (s, 9H), 0.14–0.01 (m, 6H). ^13^C NMR (101 MHz, CDCl_3_) δ 138.0, 132.4, 131.1, 128.5, 127.8, 127.8, 74.8, 73.4, 69.5, 68.2, 43.1, 39.6, 33.7, 29.1, 26.0, 23.9, 18.2, 16.0, −4.3, −4.6. HRMS (ESI, *m/z*) for C_24_H_43_O_3_Si^+^ [M+H]^+^: Calcd. 407.2976; found: 407.2977.

(3*S*,4*S*,9*R*)-9-((*tert*-butyldimethylsilyl)oxy)-1-hydroxy-4-methyldecan-3-yl (*tert*-butoxycarbonyl)-*D*-phenylalanyl-*L*-prolinate **19**:

To a solution of alcohol **18** (510 mg, 1.3 mmol, 1.0 equiv.) and acid **3** (480 mg, 1.3 mmol, 1.1 equiv.) in anhydrous THF (30 mL, 0.040 M) at 0 °C under an argon atmosphere, PPh_3_ (990 mg, 3.8 mmol, 3.0 equiv.) and DIAD (0.5 mL, 2.5 mmol, 2.0 equiv.) were added. After being stirred at room temperature for 9 h, the reaction mixture was quenched with saturated aqueous solution of NaHCO_3_ (20 mL) and extracted with EtOAc (3 × 30 mL). The combined organic layers were washed with saturated aqueous solution of brine (30 mL), dried over anhydrous Na_2_SO_4_, filtered, and concentrated in vacuo. Purification of the crude product was performed by flash chromatography on silica gel (hexanes/EtOAc = 9/1) to afford Mitsunobu product (822 mg, 87%) as a colorless oil.

To a solution of Mitsunobu product (125 mg, 0.17 mmol, 1.0 equiv.) in EtOAc (10 mL, 0.020 M), palladium on charcoal (50 mg, 10% Pd, 0.047 mmol, 0.28 equiv.) was added under argon atmosphere. The reaction flask was evacuated and purged with hydrogen three times. The reaction mixture was stirred under a hydrogen atmosphere at room temperature for 9 h; the flask was then evacuated and purged with nitrogen three times, and the catalyst was removed by filtration through a pad of celite. The filtrate was concentrated under reduced pressure to afford crude product, which was purified by flash chromatography on silica gel (hexanes/EtOAc = 4/1) to afford **19** (107 mg, 95%) as a colorless oil. TLC: R*_f_* = 0.20 (silica gel, ethyl acetate/hexanes = 1/4). UV and PMA stain. ${\left[\alpha \right]}_{D}^{24.3}$ = −38.30 (*c* 2.0, CHCl_3_). ^1^H NMR (400 MHz, CDCl_3_) δ 7.27–7.23 (m, 2H), 7.23–7.17 (m, 3H), 5.40 (d, *J* = 8.6 Hz, 1H), 5.09–4.98 (m, 1H), 4.70–4.54 (m, 1H), 4.24 (dd, *J* = 8.4, 4.1 Hz, 1H), 3.80–3.63 (m, 3H), 3.55–3.47 (m, 1H), 3.06 (dd, *J* = 12.8, 5.4 Hz, 1H), 2.91 (dd, *J* = 12.8, 9.5 Hz, 1H), 2.69–2.56 (m, 1H), 1.99–1.90 (m, 1H), 1.88–1.72 (m, 4H), 1.68–1.56 (m, 1H), 1.54–1.46 (m, 1H), 1.43 (s, 9H), 1.36–1.20 (m, 8H), 1.10 (d, *J* = 6.1 Hz, 3H), 0.92–0.84 (m, 12H), 0.04 (s, 3H), 0.03 (s, 3H). ^13^C NMR (101 MHz, CDCl_3_) δ 172.8, 170.5, 155.1, 136.6, 129.6, 128.5, 127.0, 79.9, 75.6, 68.7, 59.2, 58.8, 53.8, 47.1, 40.5, 39.8, 37.1, 34.8, 33.2, 29.2, 28.5, 27.2, 26.0, 24.7, 23.9, 18.2, 14.7, −4.3, −4.6. HRMS (ESI, *m*/*z*) for C_36_H_63_N_2_O_7_Si^+^ [M+H]^+^: Calcd. 663.4399; found: 663.4401.

(3*S*,4*R*,6*S*,7*S*,12*R*)-12-((*tert*-butyldimethylsilyl)oxy)-4-hydroxy-3,7-dimethyltridec-1-en-6-yl (*tert*-butoxycarbonyl)-*D*-phenylalanyl-*L*-prolinate **20**:

To a solution of alcohol **19** (237 mg, 0.70 mmol, 1.0 equiv.) in dry DCM (10 mL, 0.070 M) at 0 °C under argon atmosphere, Dess–Martin periodinane (353 mg, 0.83 mmol, 1.2 equiv.) was added. After being stirred at room temperature for 2 h, the reaction mixture was quenched with saturated aqueous solution of Na_2_SO_3_ (10 mL) and extracted with EtOAc (3 × 10 mL). The combined organic extracts were washed with saturated aqueous solution of copper sulfate (10 mL) and brine (10 mL), dried over anhydrous Na_2_SO_4_, filtered, and concentrated in vacuo. The residue was used directly in the next step without further purification.

To a suspension of *t*BuOK (365 mg, 3.3 mmol, 5.0 equiv.) in anhydrous THF (10 mL, 0.065 M) at −78 °C was added *trans*-2-butene (255 mg, 4.6mmol, 7.0 equiv.), followed by the dropwise addition of *n*BuLi (2.0 mL, 3.3 mmol, 1.6 M in hexane, 5.0 equiv.). After the addition of *n*BuLi, the reaction mixture was allowed to warm to −45 °C and stirred for 30 min. After being re-cooled to −78 °C, a solution of (-)-Ipc_2_BOMe (1.2 g, 3.9 mmol, 6.0 equiv.) in anhydrous THF (2 mL) was added and stirred for 30 min at −78 °C. BF_3_·Et_2_O (0.66 mL, 5.2 mmol, 8.0 equiv.) was then added followed by a solution of aldehyde in THF (2 mL). After being stirred at −78 °C for 6 h, the reaction mixture was quenched with MeOH (5 mL) and allowed to warm to room temperature. Solvent was removed in vacuo and the residue was redissolved in THF (6 mL) and H_2_O (4 mL), and NaBO_3_.4H_2_O (500 mg, 3.3 mmol, 5.0 equiv.) was then added. The mixture was stirred at room temperature for 3 h and poured into a mixture of EtOAc (30 mL) and H_2_O (10 mL). The layers were separated, and the aqueous layer was further extracted with EtOAc (2 × 10 mL). The combined organic extracts were washed with brine (10 mL), dried over anhydrous Na_2_SO_4_, filtered, and concentrated in vacuo. Purification of the crude product was performed by flash chromatography on silica (hexanes/EtOAc = 4/1) to afford two separable diastereoisomers (396 mg, 85% total yield, dr > 20:1) with **20** (colorless oil) as the major product. TLC: R*_f_* = 0.40 (silica gel, ethyl acetate/hexanes = 1/4). UV and PMA stain. ${\left[\alpha \right]}_{D}^{24.9}$ = −30.60 (*c* 1.0, CHCl_3_). ^1^H NMR (400 MHz, CDCl_3_) δ 7.31–7.14 (m, 5H), 5.86 (ddd, *J* = 16.4, 11.1, 7.6 Hz, 1H), 5.43 (d, *J* = 8.6 Hz, 1H), 5.17–5.04 (m, 3H), 4.69–4.55 (m, 1H), 4.31–4.22 (m, 1H), 3.83–3.73 (m, 1H), 3.72–3.63 (m, 1H), 3.57–3.44 (m, 1H), 3.09 (dd, *J* = 12.8, 5.3 Hz, 1H), 2.93 (dd, *J* = 12.8, 9.6 Hz, 1H), 2.69–2.53 (m, 1H), 2.40–2.28 (m, 1H), 2.04–1.81 (m, 4H), 1.80–1.72 (m, 1H), 1.70–1.59 (m, 2H), 1.47 (s, 9H), 1.39–1.30 (m, 3H), 1.32–1.22 (m, 5H), 1.17–1.05 (m, 6H), 0.95–0.81 (m, 12H), 0.10–0.02 (m, 6H). ^13^C NMR (101 MHz, CDCl_3_) δ 171.9, 170.3, 155.0, 140.3, 136.5, 129.6, 128.4, 127.0, 115.5, 79.7, 72.9, 68.7, 59.2, 53.8, 47.0, 44.3, 40.6, 39.7, 37.1, 36.3, 33.0, 29.7, 29.0, 28.4, 28.2, 27.2, 26.0, 24.5, 23.9, 18.2, 15.3, 14.4, −4.3, −4.7. HRMS (ESI, *m*/*z*) for C_40_H_69_N_2_O_7_Si^+^ [M+H]^+^: Calcd. 717.4869; found: 717.4866.

(5*R*,7*S*,8*S*,13*R*)-5-((*S*)-but-3-en-2-yl)-2,2,3,3,8,13,15,15,16,16-decamethyl-4,14-dioxa-3,15-disilaheptadecan-7-yl (*tert*-butoxycarbonyl)-*D*-phenylalanyl-*L*-prolinate **21**:

To a solution of alcohol **20** (4450 mg, 0.63 mmol, 1.0 equiv.) in dry DCM (5 mL, 0.15 M) at 0 °C under argon atmosphere, Et_3_N (200 μL, 1.3 mmol, 2.0 equiv.) was added, followed by TBSOTf (220 μL, 0.95 mmol, 1.5 equiv.). The reaction mixture was warmed up to ambient temperature and stirred for 1 h, and then quenched with a saturated aqueous solution of NaHCO_3_ (5 mL). The aqueous layer was extracted with EtOAc (2 × 5 mL). The combined organic layers were washed with H_2_O (10 mL) and brine (10 mL), dried over anhydrous Na_2_SO_4_, filtered, and concentrated in vacuo. Purification of the crude product was performed by flash chromatography on silica (hexanes/EtOAc = 9/1) to afford silyl ether **21** (490 mg, 94%) as a colorless oil. TLC: R*_f_* = 0.40 (silica gel, ethyl acetate/hexanes = 1/9). UV and PMA stain. ${\left[\alpha \right]}_{D}^{25.8}$ = −35.40 (*c* 1.0, CHCl_3_). ^1^H NMR (400 MHz, CDCl_3_) δ 7.29–7.18 (m, 5H), 5.81 (ddd, *J* = 17.0, 10.5, 8.4 Hz, 1H), 5.39 (d, *J* = 8.4 Hz, 1H), 5.10–4.98 (m, 2H), 4.97–4.90 (m, 1H), 4.71–4.57 (m, 1H), 4.31 (dd, *J* = 8.2, 3.6 Hz, 1H), 3.88–3.68 (m, 2H), 3.56–3.44 (m, 1H), 3.08 (dd, *J* = 12.7, 5.4 Hz, 1H), 2.95 (dd, *J* = 12.7, 9.3 Hz, 1H), 2.71–2.58 (m, 1H), 2.48–2.35 (m, 1H), 2.03–1.90 (m, 1H), 1.90–1.79 (m, 4H), 1.73–1.65 (m, 2H), 1.46 (s, 9H), 1.39–1.23 (m, 8H), 1.13 (d, *J* = 6.1 Hz, 3H), 1.05 (d, *J* = 6.8 Hz, 3H), 0.93 (s, 9H), 0.91 (s, 9H), 0.85 (d, *J* = 6.9 Hz, 3H), 0.12 (s, 6H), 0.07 (s, 3H), 0.06 (s, 3H). ^13^C NMR (101 MHz, CDCl_3_) δ 171.7, 169.8, 155.0, 140.3, 136.7, 129.6, 128.4, 126.9, 115.1, 79.6, 75.0, 72.2, 68.7, 59.1, 53.6, 46.6, 42.1, 40.6, 39.7, 37.0, 36.6, 33.0, 29.2, 28.4, 27.3, 26.0, 24.5, 23.8, 18.2, 18.1, 17.3, 14.0, −4.3, −4.3, −4.6, −4.6. HRMS (ESI, *m*/*z*) for C_46_H_83_N_2_O_7_Si_2_^+^ [M+H]^+^: Calcd. 831.5733; found: 831.5735.

(3*S*,4*R*,6*S*,7*S*,12*R*)-12-((*tert*-butyldimethylsilyl)oxy)-4-hydroxy-3,7-dimethyltridec-1-en-6-yl (*tert*-butoxycarbonyl)-*D*-phenylalanyl-*L*-prolinate **22**:

In a solution of alkene **20** (441 mg, 0.53 mmol, 1.0 equiv.) in DCM at −78 °C, ozone was bubbled through until the solution became slightly blue. PPh_3_ (700 mg, 2.65 mmol, 5.0 equiv.) was added and the resultant solution was stirred at room temperature for 1 h. After concentration, the residue was used directly in the next step without further purification.

To a solution of the above crude aldehyde and PhI(OAc)_2_ (512 mg, 1.6 mmol, 3.0 equiv.) in DCM (20 mL, 0.026 M) at 0 °C, TEMPO (33 mg, 0.21 mmol, 0.40 equiv.), and H_2_O (2 mL) were added. The reaction mixture was warmed up to ambient temperature and stirred for 12 h, and then quenched with a saturated aqueous solution of Na_2_SO_3_ (10 mL). The aqueous layer was extracted with EtOAc (3 × 10 mL), and the combined organic layers were washed with H_2_O (10 mL) and brine (10 mL), dried over anhydrous Na_2_SO_4_, filtered. and concentrated in vacuo. Purification of the crude product was performed by flash chromatography on silica (hexanes/EtOAc = 4/1) to afford acid **22** (306 mg, 68%) as a colorless oil. TLC: R*_f_* = 0.40 (silica gel, ethyl acetate/hexanes = 1/4). UV and PMA stain. ${\left[\alpha \right]}_{D}^{26.9}$ = −45.40 (*c* 1.0, CHCl_3_). ^1^H NMR (400 MHz, CDCl_3_) δ 7.30–7.10 (m, 5H), 5.32 (dd, *J* = 8.7, 2.2 Hz, 1H), 5.15–4.96 (m, 1H), 4.75–4.55 (m, 1H), 4.22 (dd, *J* = 8.3, 4.0 Hz, 1H), 4.10 (dt, *J* = 8.4, 4.3 Hz, 1H), 3.76 (q, *J* = 5.9 Hz, 1H), 3.65–3.43 (m, 1H), 3.05 (dd, *J* = 12.9, 5.4 Hz, 1H), 2.96–2.81 (m, 2H), 2.79–2.62 (m, 1H), 2.02–1.74 (m, 4H), 1.70–1.60 (m, 1H), 1.60–1.51 (m, 1H), 1.44 (s, 9H), 1.37–1.24 (m, 8H), 1.21 (d, *J* = 7.0 Hz, 3H), 1.11 (d, *J* = 6.1 Hz, 3H), 0.93 (s, 9H), 0.91–0.81 (m, 12H), 0.15 (d, *J* = 5.0 Hz, 6H), 0.05 (s, 3H), 0.04 (s, 3H). ^13^C NMR (101 MHz, CDCl_3_) δ 177.0, 171.3, 170.3, 155.0, 136.4, 129.5, 128.4, 127.0, 79.8, 77.4, 74.5, 71.3, 68.6, 59.1, 53.6, 46.9, 44.2, 40.3, 39.7, 36.8, 35.9, 32.9, 29.1, 28.4, 27.3, 26.0, 25.8, 24.5, 23.9, 23.8, 18.2, 17.9, 14.4, 13.8, −4.4, −4.4, −4.7, −5.0. HRMS (ESI, *m/z*) for C_45_H_81_N_2_O_9_Si_2_^+^ [M+H]^+^: Calcd. 849.5475; found: 849.5479.

Acremolide A (**1**):

To a solution of **22** (77 mg, 0.091 mmol, 1.0 equiv.) in DCM (5 mL, 0.020 M) at 0 °C, Et_3_N (0.25 mL, 1.8 mmol, 20.0 equiv.) was added, followed by dropwise addition of trimethylsilyl trifluoromethanesulfonate (0.41 mL, 1.8 mmol, 20.0 equiv.). The reaction mixture was allowed to warm for 1 h at room temperature and then quenched with saturated aqueous solution of NaHCO_3_ (10 mL) and extracted with EtOAc (3 × 10 mL). The combined organic layers were washed with saturated aqueous solution of NH_4_Cl (10 mL), brine (10 mL), dried over anhydrous Na_2_SO_4_, filtered and concentrated in vacuo. The residue was used directly in the next step without further purification.

To a solution of the above crude amine in DCM (90 mL, 0.001 M) at 0 °C was added DIPEA (0.16 mL, 0.91 mmol, 10.0 equiv.), HATU (173 mg, 0.46 mmol, 5.0 equiv.) and HOAt (37 mg, 0.27 mmol, 3.0 equiv.). The reaction mixture was allowed to warm for 9 h at room temperature and then concentrated in vacuo and the residue was redissolved in EtOAc (30 mL) and quenched with 4% aqueous citric acid solution. The aqueous layer was extracted with EtOAc (3 × 20 mL), and the combined organic layers were washed with saturated aqueous solution of NaHCO_3_ (15 mL) and brine (15 mL), dried over anhydrous Na_2_SO_4_, filtered, and concentrated in vacuo. Purification of the crude product was performed by flash chromatography on silica gel (hexanes/EtOAc = 2/1) to afford macrocyclization product (399 mg, 60%) as a colorless oil.

To a solution of macrocyclization product (16 mg, 0.028 mmol, 1.0 equiv.) in THF (4 mL, 0.007 M) at 0 °C was added TBAF (2.0 mL, 2.0 mmol, 100 equiv., 1.0 M in THF). The reaction mixture was warmed up to ambient temperature and stirred for 6 h, and then quenched with a saturated aqueous solution of NH_4_Cl (10 mL). The aqueous layer was extracted with EtOAc (3 × 10 mL). The combined organic layers were washed with H_2_O (10 mL) and brine (10 mL), dried over anhydrous Na_2_SO_4_, filtered, and concentrated in vacuo. Purification of the crude product was performed by flash chromatography on silica (hexanes/EtOAc = 4/1) to afford acremolide A (**1**) (8.0 mg, 57%) as a colorless oil. TLC: R*_f_* = 0.40 (silica gel, ethyl acetate/hexanes = 4/1). UV and PMA stain. ${\left[\alpha \right]}_{D}^{24.8}$ = −109 (*c* 0.05, MeOH). Major *cis* conformer: ^1^H NMR (400 MHz, DMSO-*d*_6_) δ 8.23 (d, *J* = 8.9 Hz, 1H), 7.29–7.16 (m, 5H), 5.01 (dd, *J* = 8.5, 3.0 Hz, 1H), 4.59–4.47 (m, 2H), 4.28 (dd, *J* = 6.9, 4.6 Hz, 1H), 3.76–3.67 (m, 1H), 3.64–3.51 (m, 2H), 3.53–3.41 (m, 1H), 3.21 (dd, *J* = 13.8, 3.9 Hz, 1H), 2.83 (dd, *J* = 13.8, 11.0 Hz, 1H), 2.33 (d, *J* = 7.3 Hz, 1H), 2.32–2.20 (m, 1H), 1.96–1.85 (m, 2H), 1.84–1.75 (m, 2H), 1.75–1.71 (m, 1H), 1.71–1.65 (m, 1H), 1.61–1.57 (m, 1H), 1.53–1.48 (m, 1H), 1.27–1.17 (m, 4H), 1.02 (d, *J* = 6.2 Hz, 3H), 0.83 (d, *J* = 6.3 Hz, 3H), 0.73 (d, *J* = 6.7 Hz, 3H). ^13^C NMR (101 MHz, DMSO-*d*_6_) δ 175.9, 171.4, 169.0, 137.6, 129.0, 127.9, 126.2, 75.3, 69.4, 65.5, 57.8, 55.0, 48.6, 41.8, 38.6, 35.3, 34.9, 31.7, 31.3, 25.4, 23.5, 20.5, 15.4, 15.2. HRMS (ESI, *m*/*z*) for C_28_H_42_N_2_O_6_Na^+^ [M+Na]^+^: Calcd. 525.2936; found 525.2935.

## 4. Conclusions

The absolute stereochemical configurations of acremolides A and B were predicted based on the biochemistry-based rule, which was validated through total syntheses. The features of the syntheses include a sequential Krische’s Ir-catalyzed crotylation and Brown’s borane-mediated crotylation to establish the four stereogenic centers in the fatty acid substructure, a cross-metathesis reaction to link two fragments efficiently, and a Mitsunobu esterification reaction followed by a macrolactamization reaction to form the 12-membered ring. The total syntheses of acremolides A and B were achieved in 12 and 10 linear steps, with overall yields of 8.3% and 19%, respectively.

## Data Availability

Data are contained within the article and Supplementary Materials.

## Supplementary Materials

The following supporting information can be downloaded at: https://www.mdpi.com/article/10.3390/molecules29153599/s1, Copies of NMR spectra (^1^H and ^13^C) of **1**, **2**, **9**, **13**, **15**, **16**, **18**–**22**.
