# Supplementary material for: Total Syntheses and Stereochemical Assignment of Acremolides A and B
Source: Molecules. 2024 Jul 30;29(15):3599. doi: 10.3390/molecules29153599 (PMC11314444; doi:10.3390/molecules29153599)

Supporting Information

Total Syntheses and Stereochemical Assignment of Acremolides A and B

Yi Xiao†, Junyang Liu†, Yangyang Jiang, Yian Guo*, and Tao Ye,*

Content

Proposed biosynthetic pathway of acremolides A and B S2
Table S1: Comparison of ^{1}H NMR Data of natural and synthetic acremolide A S3
Table S2: Comparison of ^{13}C NMR Data of natural and synthetic acremolide A S5
Table S3: Comparison of ^{1}H NMR Data of natural and synthetic acremolide B S6
Table S4: Comparison of ^{13}C NMR Data of natural and synthetic acremolide B S8
NMR Spectra S9

Proposed biosynthetic pathway of acremolides A and B

Plausible biosynthesis of acremolides A and B is proposed and shown in Scheme S1. According to the Biochemistry-guided rules related to the prediction of stereochemical configuration fungal polyketides, the previously unknown stereostructure of acremolides A and B could also be represented as illustrated in Scheme S1. In short, the ketoreductase (KR) domain reduces the corresponding keto group, yielding a $°R_{KR}$ configuration (i. label the group on the PK chain elongation side with R_C; ii. label the group on the PK chain initial side with R_M; iii. priority: OH > R_C > R_M > H; iv. observe the stereocenter from the opposite of H, the group with the lowest priority, and connect other 3 groups according to their priorities in descending order; v. the connection should be clockwise) of the hydroxyl group at C11, C5, and C3 (marked in red). Alternatively, the enoyl reductase domain reduces enoyl moieties, resulting in a $°S_{ER}$ configuration of the methyl group at C6 (marked in green). Additionally, the methyltransferase (MT) domain adds a methyl group at the α position, producing an $°R_{MT}$ configuration of the methyl group at C2 (marked in blue). We have added this scheme in the supporting information accordingly.

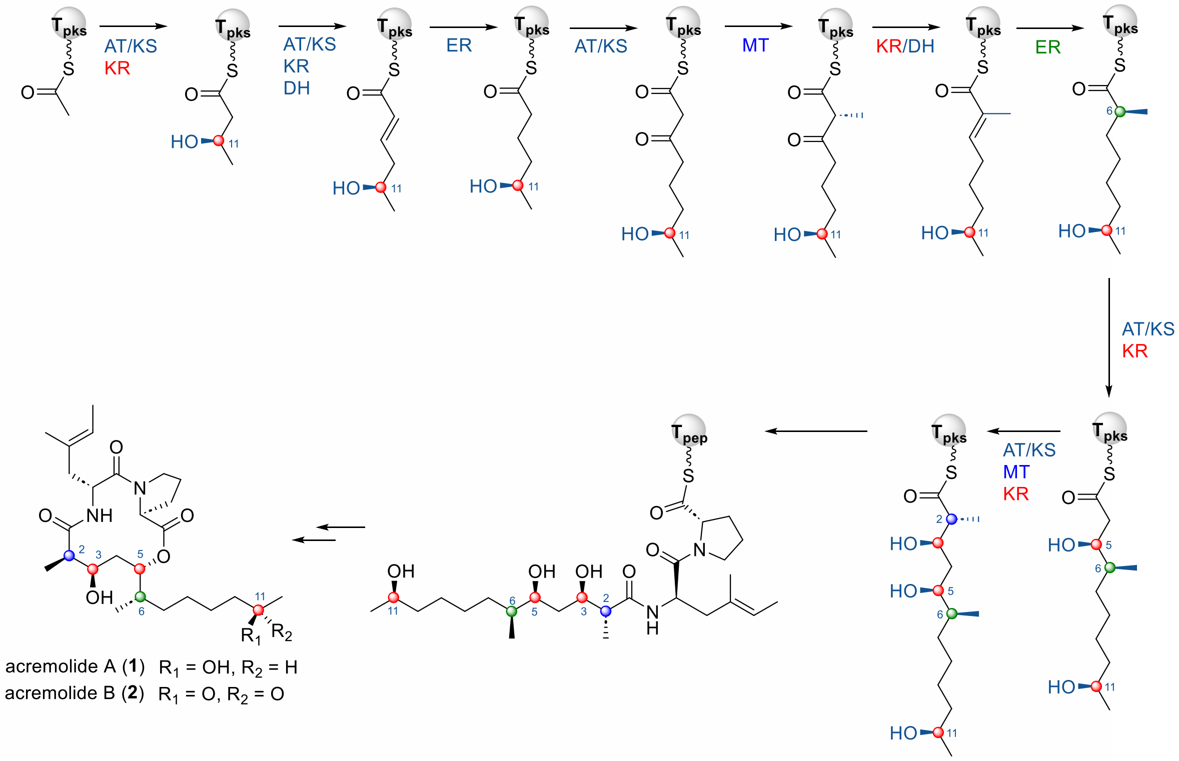

Scheme S1. Plausible biosynthetic pathway of acremolides A and B

Table S1: Comparison of ^{1}H NMR Data of natural and synthetic acremolide A

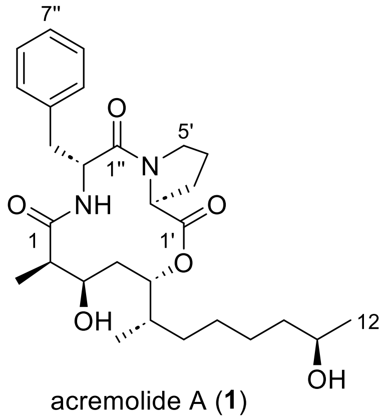

acremolide A (**1**)

**No.**	**acremolide A**				
	**Natural *cis* {δ_1, ppm (mult., *J*, Hz)}**	**Natural *trans* {$\delta_{1'}$, ppm (mult., *J*, Hz)}**	**Synthetic *cis* {δ_2, ppm (mult., *J*, Hz)}**	**Synthetic *trans* {$\delta_{2'}$, ppm (mult., *J*, Hz)}**	**$\Delta_{cis} = \delta_1 - \delta_2$; $\Delta_{trans} = \delta_{1'} - \delta_{2'}$ (ppm)**
**2**	2.34(dq, 7.0, 1.3)	2.57(dq, 7.0, 3.3)	2.33(overlap)	2.57(dd, 6.8, 3.1)	0.01; 0
**3**	3.72(m)	3.93(m)	3.72(m)	3.93(m)	0; 0
**4a**	1.84(ddd, 15.0, 6.4, 1.9)	1.51(m)	1.84(overlap)	1.51(m)	0; 0
**4b**	1.73(ddd, 15.0, 7.0, 3.7)		1.73(dd, 6.6, 3.9)		0; 0
**5**	4.55(m)	4.61(ddd, 9.9, 2.8, 3.3)	4.55(m)	4.61(d, 8.0, 9.8)	0; 0
**6**	1.69(m)	1.94(m)	1.69(m)	1.94(m)	0; 0
**7a**	1.57(m)	1.34(m)	1.57(m)	1.34(m)	0; 0
**7b**	1.53(m)	1.03(m)	1.53(m)	1.03	0; 0
**8**	#	#	#	#	/
**9a**	1.24(m)	1.24(m)	1.24(m)	1.24(m)	0; 0
**9b**	1.02(m)	1.02(m)	1.02	1.02	0; 0
**10a**	1.33(m)	1.33(m)	1.33(m)	1.33(m)	0; 0
**10b**	1.26(m)	1.26(m)	1.26(m)	1.26(m)	0; 0
**11**	3.56(m)	3.55(m)	3.56(m)	3.55(m)	0; 0
**12**	1.02(d, 6.1)	1.02(d, 6.0)	1.02(d, 6.2)	1.02(d, 6.2)	0; 0
**2-Me**	0.73(d, 7.0)	0.83(d, 7.0)	0.73(d, 7.2)	0.83	0; 0
**6-Me**	0.83(d, 6.6)	0.73(d, 7.7)	0.83(d, 6.2)	0.73(d, 7.2)	0; 0
**3-OH**		4.41(d, 4.6)		4.40(d, 6.0)	0.01
**11-OH**	4.29(d, 4.6)	4.27(d, 4.6)	4.29(d, 4.7)	4.27(d, 4.6)	0; 0

**2′**	5.00(brd, 2.8)	4.83(brd, 6.9)	5.01(dd, 8.5, 3.0)	4.84(d, 6.2)	-0.01; -0.01
**3a′**	2.25(m)	2.18(ddd, 12.0, 11.2, 5.7)	2.25(m)	2.18(d, 5.7)	0; 0
**3b′**	1.89(m)	1.59(m)	1.89(m)	1.59(m)	0; 0
**4a′**	1.78(m)	1.77(m)	1.78(m)	1.77(m)	0; 0
**4b′**	1.90(m)	1.60(m)	1.90(m)	1.59(m)	0; 0
**5a′**	3.58(brdd, 12.0, 7.8))	3.87(ddd, 9.5, 9.0, 2.2)	3.58(m)	3.86(m)	0; 0.01
**5b′**	3.47(ddd, 12.0, 7.8, 7.6)	3.13(brdd, 9.0, 8.8)	3.47(dt, 11.9, 7.7)	3.13(m)	0; 0
**2″**	4.53(dt, 13.7, 3.8)	4.73(ddd, 9.1, 8.1, 8.0)	4.53(m)	4.73(q, 8.2)	0; 0
**3a″**	3.20(dd, 13.9, 3.8)	2.87(dd, 13.5, 8.0)	3.21(dd, 13.9, 3.9)	2.87(m)	-0.01; 0
**3b″**	2.82(dd, 13.9, 13.7)	2.82(ddd, 13.5, 8.0)	2.83(dd, 13.8, 10.9)	2.83(dd, 13.8, 10.9)	-0.01; -0.01
**4″**					
**5″**	7.20(d, 7.5)	7.26(d, 7.0)	7.21(d, 7.2)	7.26(d, 7.0)	-0.01; 0
**6″**	7.16-7.28(m)	7.16-7.28(m)	7.16-7.28(m)	7.16-7.28(m)	0; 0
**7″**	7.16-7.28(m)	7.16-7.28(m)	7.16-7.28(m)	7.16-7.28(m)	0; 0
**8″**	7.16-7.28(m)	7.16-7.28(m)	7.16-7.28(m)	7.16-7.28(m)	0; 0
**9″**	7.20(d, 7.5)	7.26(d, 7.0)	7.20(d, 7.5)	7.26(d, 7.0)	0;0
**NH-2″**	8.22(d, 8.7)	8.43(d, 9.1)	8.23(d, 8.9)	8.43(d, 9.2)	-0.01; 0

Extensive overlap of the corresponding ^{1}H NMR signals, hard to be assigned.

Table S2: Comparison of ^{13}C NMR Data of natural and synthetic acremolide A

No.	acremolide A				
	Natural *cis* (δ_1, ppm)	Natural *trans* ($\delta_{1'}$, ppm)	Synthetic *cis* (δ_2 ppm)	synthetic *trans* ($\delta_{2'}$, ppm)	$\Delta_{cis} = \delta_1 - \delta_2$; $\Delta_{trans} = \delta_{1'} - \delta_{2'}$ (ppm)
**1**	175.9	173.5	175.9	173.4	0; 0.1
**2**	41.9	46.5	41.8	46.4	0.1; 0.1
**3**	69.1	67.5	69.4	67.4	-0.3; 0.1
**4**	35.4	32.8	35.3	32.7	0.1; 0.1
**5**	75.4	77.2	75.3	77.1	0.1; 0.1
**6**	34.6	33.6	34.9	33.5	-0.3; 0.1
**7**	31.8	31.2	31.7	31.1	0.1; 0.1
**8**	#	#	#	#	/
**9**	25.5	25.5	25.4	25.4	0.1; 0.1
**10**	38.9	38.9	38.9	38.9	0; 0
**11**	65.6	65.7	65.5	65.6	0.1; 0.1
**12**	23.6	23.6	23.5	23.5	0.1; 0.1
**2-Me**	15.5	7.2	15.4	7.1	0.1; 0.1
**6-Me**	15.4	15.3	15.4	15.2	0; 0.1
**1′**	171.5	170.5	171.4	170.4	0.1; 0.1
**2′**	57.5	56.4	57.8	56.3	-0.3; 0.1
**3′**	31.5	26.3	31.3	26.2	0.2; 0.1
**4′**	20.6	23.2	20.5	23.1	0.1; 0.1
**5′**	48.6	45.4	48.6	45.3	0; 0.1
**1″**	169.1	169.8	169.0	169.7	0.1; 0.1
**2″**	55.1	52.1	55.0	52.0	0.1; 0.1
**3″**	38.7	35.2	38.6	35.3	0.1; 0.1
**4″**	137.8	137.9	137.6	137.8	0.2; 0.1
**5″**	129.1	129.1	129.0	129.0	0.1; 0.1
**6″**	127.9	128.2	127.9	128.1	0; 0.1
**7″**	126.3	126.3	126.2	126.2	0.1; 0.1
**8″**	127.9	128.2	127.9	128.1	0; 0.1
**9″**	129.1	129.1	129.0	129.0	0.1; 0.1

Table S3: Comparison of ^{1}H NMR Data of natural and synthetic acremolide B

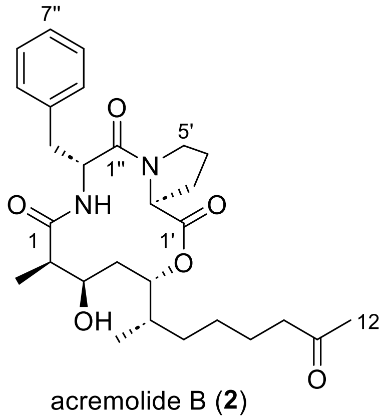

acremolide B (**2**)

No.	acremolide B				
	Natural *cis* {δ_1, ppm (mult., *J*, Hz)}	Natural *trans* {$\delta_{1'}$, ppm (mult., *J*, Hz)}	Synthetic *cis* {δ_2, ppm (mult., *J*, Hz)}	Synthetic *trans* {$\delta_{2'}$, ppm (mult., *J*, Hz)}	$\Delta_{cis} = \delta_1$-δ_2 ; $\Delta_{trans} = \delta_{1'}$-$\delta_{2'}$ (ppm)
**2**	2.32	2.57	2.33	2.58	-0.01; -0.01
**3**	3.73	3.92	3.74	3.93	0.01; -0.01
**4a**	1.82	1.50	1.82	1.50	0; 0
**4b**	1.72		1.72		0; 0
**5**	4.52	4.60(ddd, 9.8, 3.0, 2.7)	4.52	4.61(d, 9.8)	0; -0.01
**6**	1.67	1.93	1.67	1.93	0; 0
**7a**	1.18	1.01	1.18	1.01	0; 0
**7b**					
**8a**	#	#	#	#	/
**8b**					
**9a**	1.43	1.43	1.43	1.43	0; 0
**9b**	1.38	1.38	1.38	1.38	0; 0
**10**	2.40(brt, 7.5)	2.40(brt, 7.5)	2.41(t, 7.6)	2.41(t, 7.6)	-0.01; -0.01
**12**	2.06(d, 5.7)	2.06(d, 5.7)	2.07(d, 3.8)	2.05(d, 3.2)	-0.01; 0.01
**2-Me**	0.73(d, 7.3)	0.83(d, 6.7)	0.73(d, 7.2)	0.83	0; 0
**6-Me**	0.83(d, 6.7)	0.73(d, 7.3)	0.83(d, 6.3)	0.73	0; 0
**2′**	5.00(dd, 8.2, 2.8)	4.83(brd, 7.0)	5.00(dd, 8.4, 3.1)	4.84(d, 6.3)	0; -0.01
**3a′**	2.26	2.18	2.26	2.18	0; 0
**3b′**	1.91	1.58	1.91	1.58	0; 0
**4a′**	1.90	1.77	1.90	1.77	0; 0
**4b′**	1.78	1.61	1.78	1.61	0; 0

**5a′**	3.58(ddd, 12.0, 7.8, 4.7))	3.87 (ddd, 11.8, 9.3, 3.0)	3.59	3.87	-0.01; 0
**5b′**	3.47 (ddd, 12.0, 7.7, 7.6)	3.13	3.47	3.14	0; -0.01
**2′′**	4.54	4.73 (dd, 8.0, 7.8)	4.53	4.72	0.01; 0.01
**3a′′**	3.20(dd, 13.8, 3.7)	3.02(dd, 13.0, 8.0)	3.21(dd, 13.8, 3.8)	3.03(dd, 13.3, 7.8)	-0.01; -0.01
**3b′′**	2.81(dd, 13.8, 11,1)	2.87	2.83(dd, 13.7, 10.6)	2.89	-0.02; -0.02
**5′′**	7.21	7.25	7.21	7.25	0; 0
**6′′**	7.16-7.21	7.16-7.21	7.16-7.21	7.16-7.21	0; 0
**7′′**	7.16-7.21	7.16-7.21	7.16-7.21	7.16-7.21	0; 0
**8′′**	7.16-7.21	7.16-7.21	7.16-7.21	7.16-7.21	0; 0
**9′′**	7.21	7.25	7.21	7.25	0; 0
**NH-2′′**	8.22(brd, 8.4)	8.44(brd, 9.1)	8.20(t, 9.7)	8.45(d, 9.2)	0.02; -0.01

Extensive overlap of the corresponding ^{1}H NMR signals, hard to be assigned.

Table S4: Comparison of ^{13}C NMR Data of natural and synthetic acremolide B

#

No.	acremolide B				
	Natural *cis* (δ_1, ppm)	Natural *trans* ($\delta_{1'}$, ppm)	Synthetic *cis* (δ_2 ppm)	synthetic *trans* ($\delta_{2'}$, ppm)	$\Delta_{cis} = \delta_1 - \delta_2$; $\Delta_{trans} = \delta_{1'} - \delta_{2'}$ (ppm)
**1**	175.7	173.0	175.6	173.3	0.1; -0.3
**2**	41.7	46.2	41.7	46.2	0; 0
**3**	69.2	67.4	69.1	67.3	0.1; 0.1
**4**	35.1	36.3	35.1	/	0; /
**5**	75.3	77.0	75.1	76.9	0.2; 0.1
**6**	34.7	33.4	34.7	33.1	0; 0.3
**7**	30.4	31.0	30.7	31.0	-0.3;0
**8**	#	#	#	#	/
**9**	#	#	#	#	/
**10**	42.4	42.4	42.4	42.4	0; 0
**11**	208.0	208.1	208.1	208.3	-0.1; -0.2
**12**	29.4	29.4	29.3	29.4	0.1; 0
**2-Me**	15.1	6.8	15.1	6.8	0; 0
**6-Me**	15.0	15.1	15.0	15.1	0; 0
**1′**	171.2	170.8	171.2	170.9	0; -0.1
**2′**	57.6	56.2	57.7	56.1	-0.1; 0.1
**3′**	31.3	25.9	31.2	26.0	0.1; -0.1
**4′**	20.2	22.9	20.3	22.9	-0.1; 0
**5′**	48.5	45.1	48.4	45.1	0.1; 0
**1″**	168.8	169.4	168.9	169.6	-0.1; -0.2
**2″**	54.9	52.0	54.9	51.8	0; 0.2
**3″**	38.5	34.8	38.4	34.8	0.1; 0
**4″**	137.5	137.9	137.5	137.7	0; 0.2
**5″**	128.9	128.8	128.8	128.8	0.1; 0
**6″**	#	#	#	#	/
**7″**	#	126.0	#	126.0	0; 0
**8″**	#	#	#	#	/
**9″**	128.9	128.8	128.8	128.8	0.1; 0

Extensive overlap of the corresponding ^{13}C NMR signals, hard to be assigned.

NMR Spectra

¹H NMR Spectra for **9** (400 MHz, $CDCl_3$)

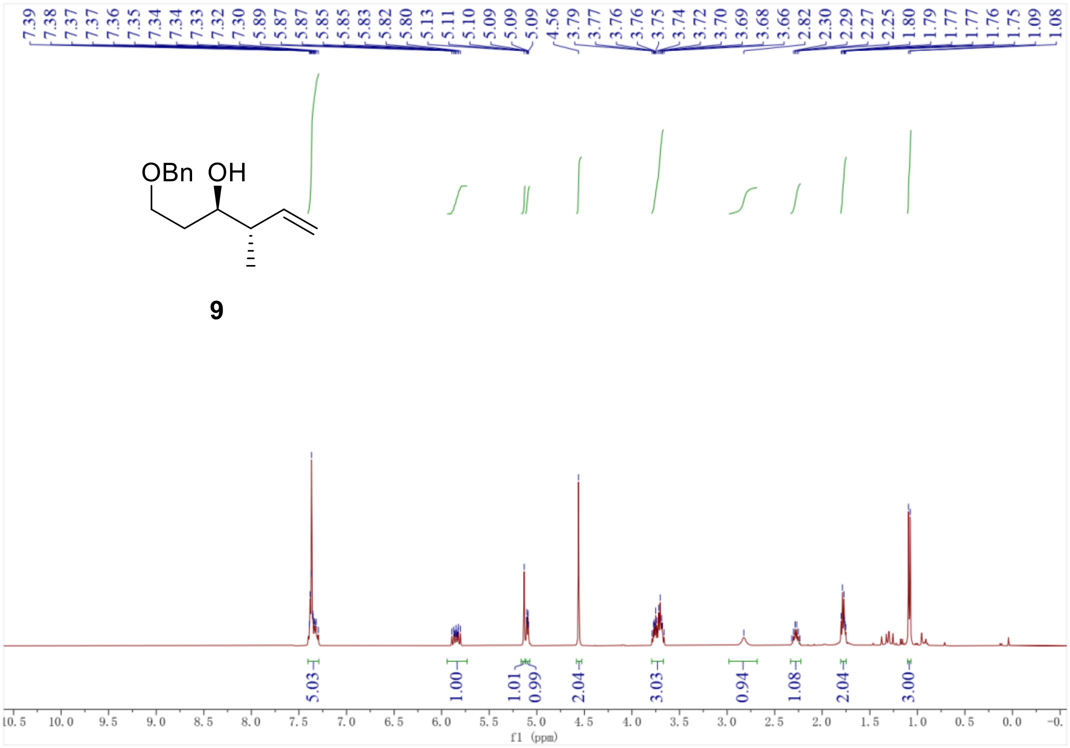

¹³C NMR Spectra for **9** (101 MHz, $CDCl_3$)

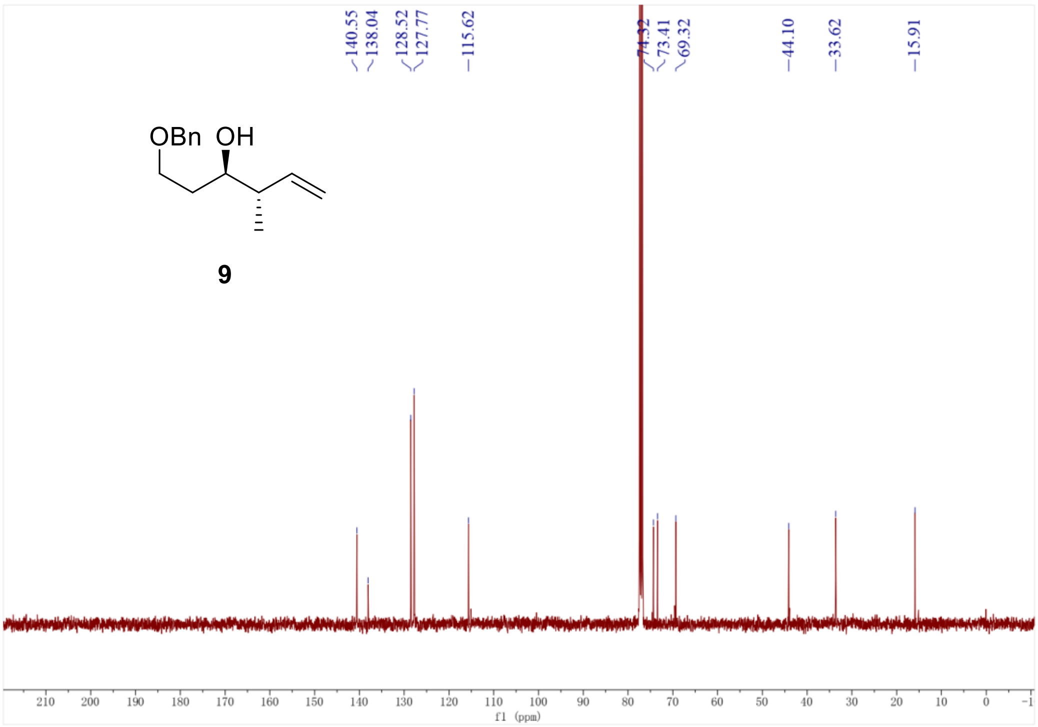

^{1}H NMR Spectra for **10** (400 MHz, $CDCl_3$)

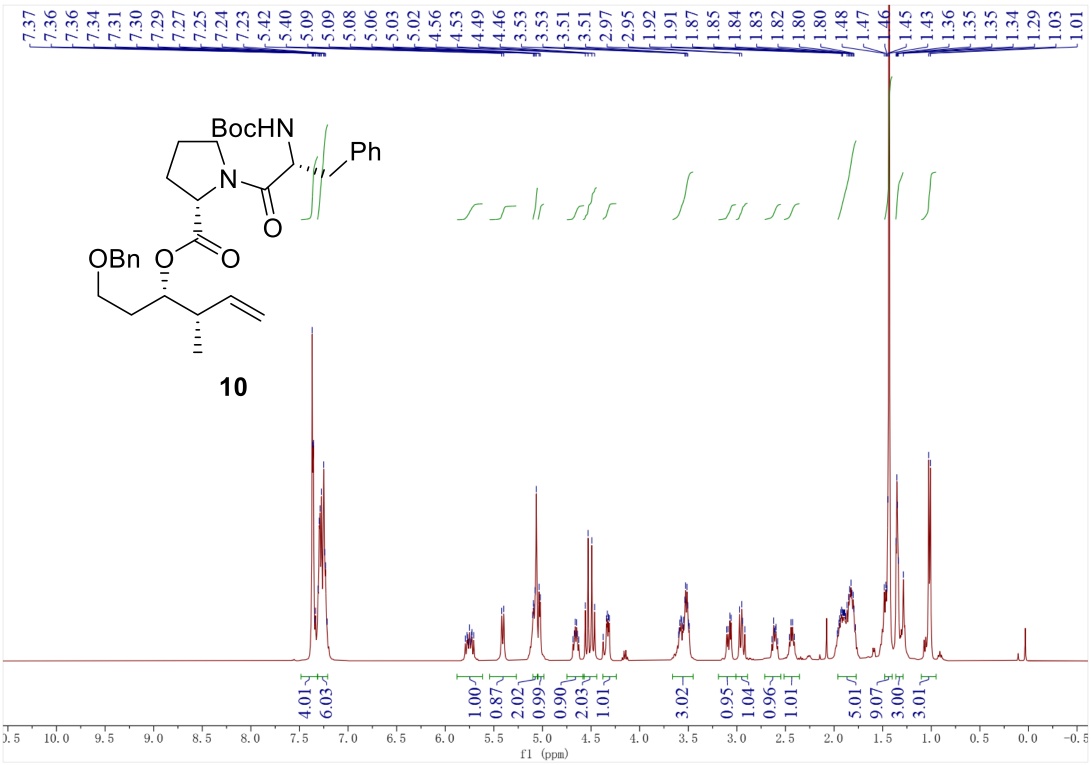

^{13}C NMR Spectra for **10** (101 MHz, $CDCl_3$)

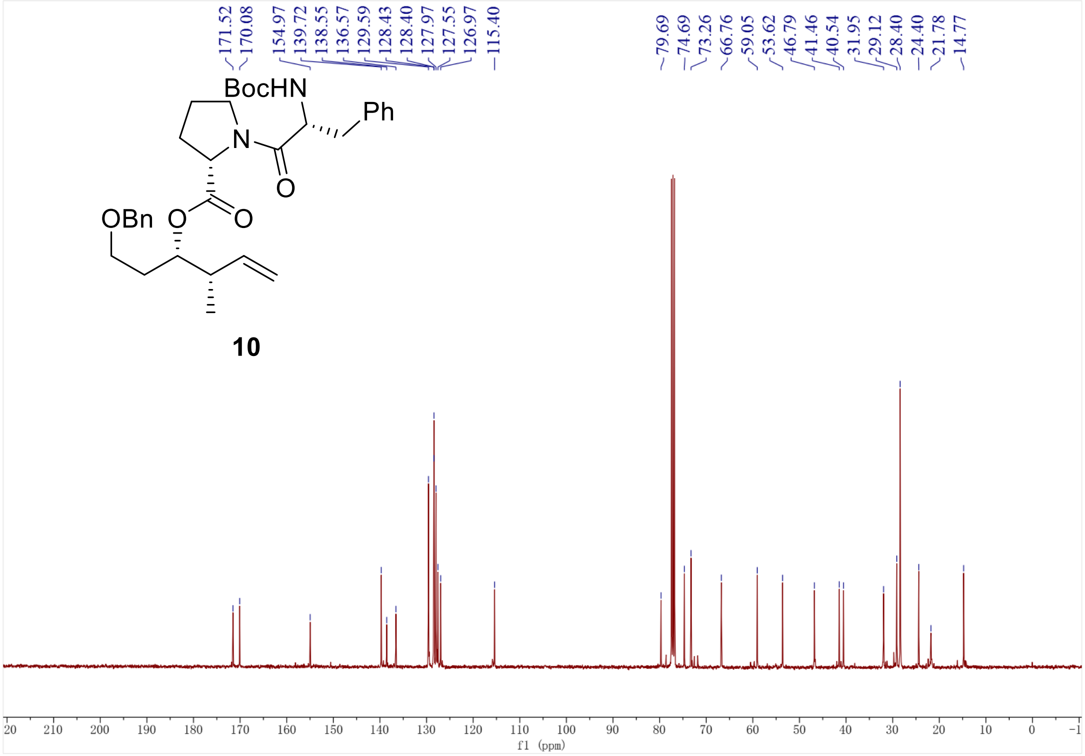

^{1}H NMR Spectra for **11** (400 MHz, $CDCl_3$)

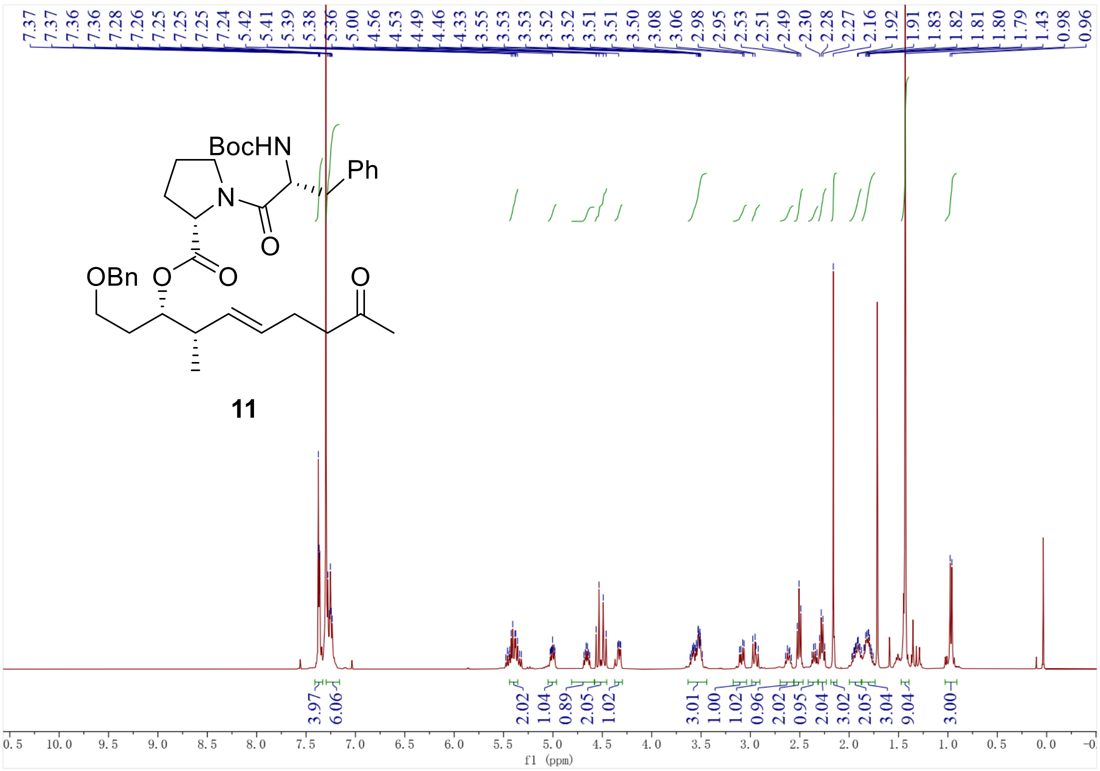

^{13}C NMR Spectra for **11** (101 MHz, $CDCl_3$)

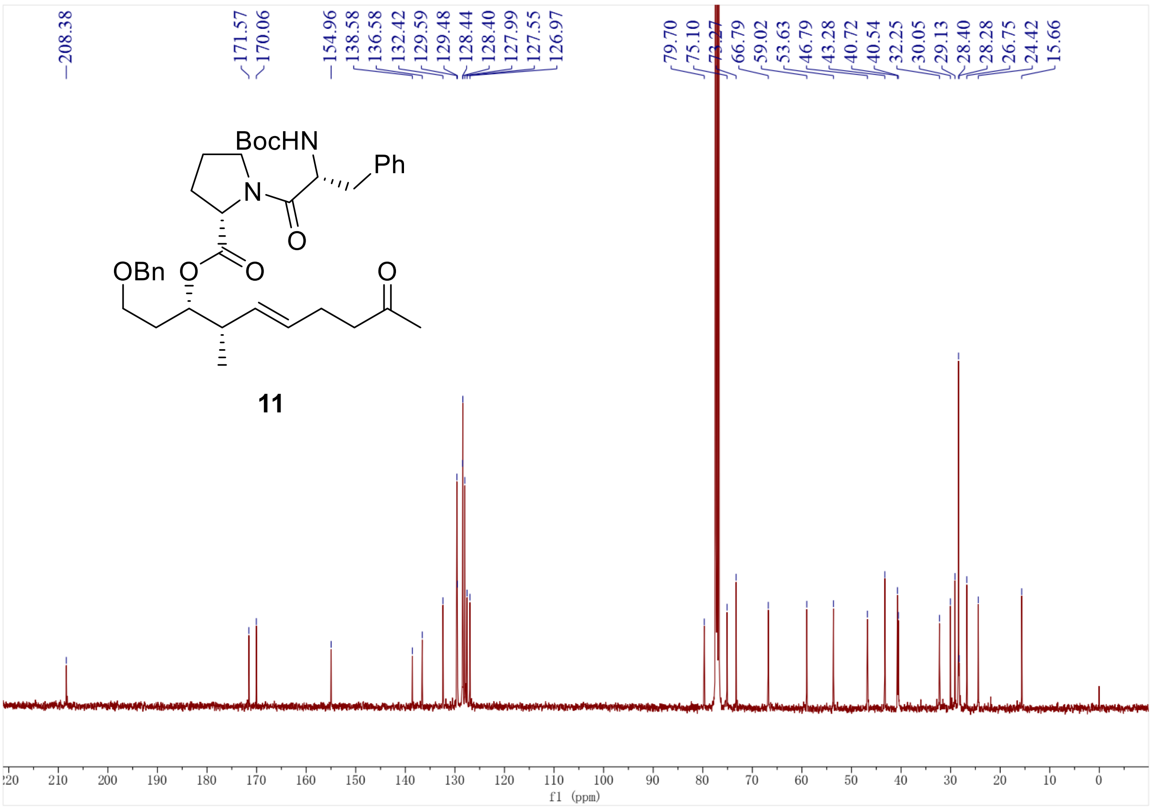

^{1}H NMR Spectra for **12** (400 MHz, $CDCl_3$)

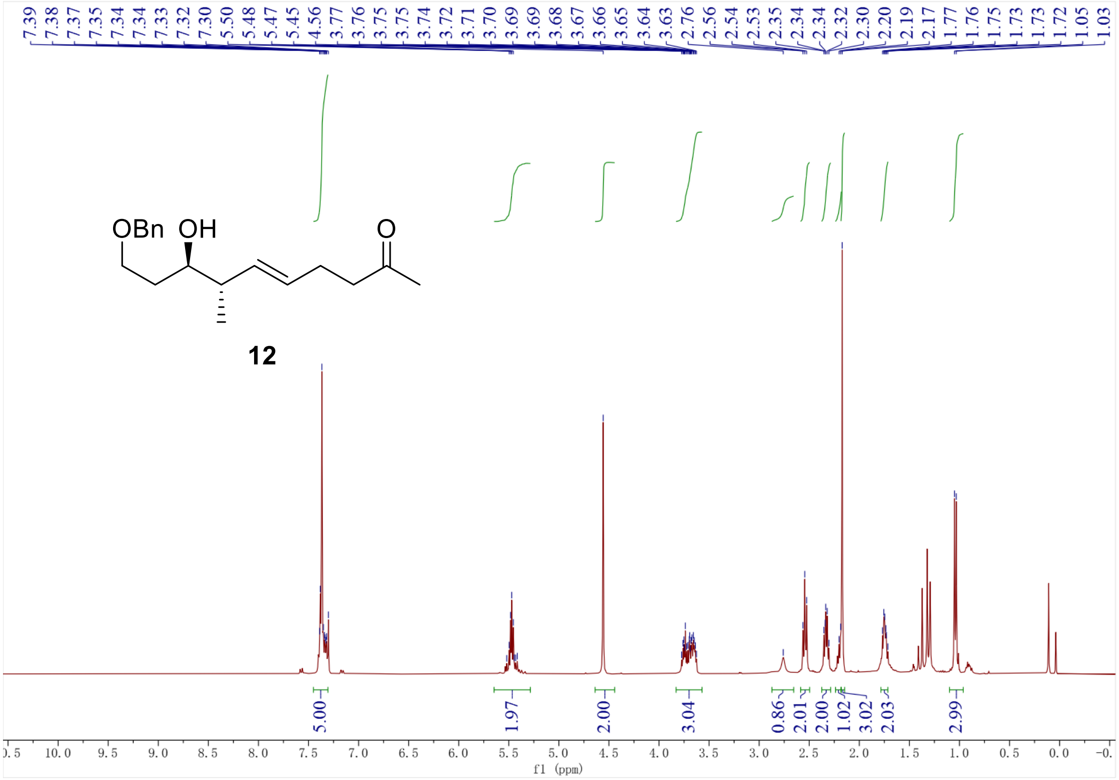

^{13}C NMR Spectra for **12** (101 MHz, $CDCl_3$)

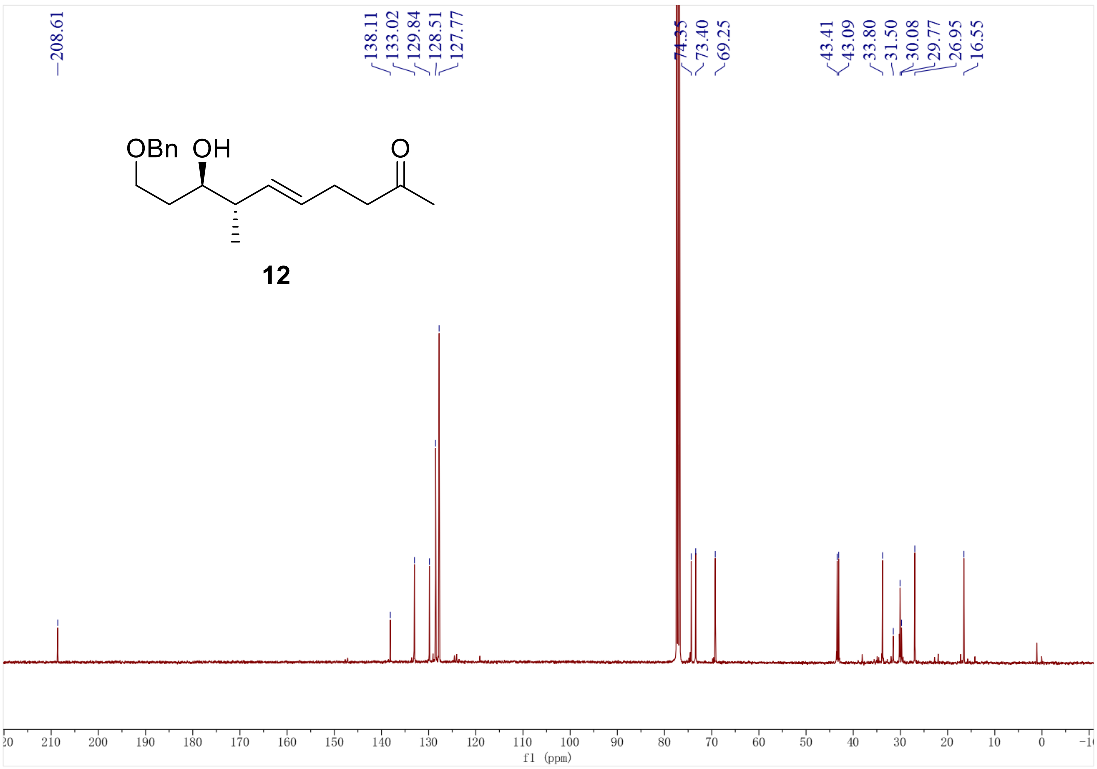

^{1}H NMR Spectra for **13** (400 MHz, CDCl$_3$)

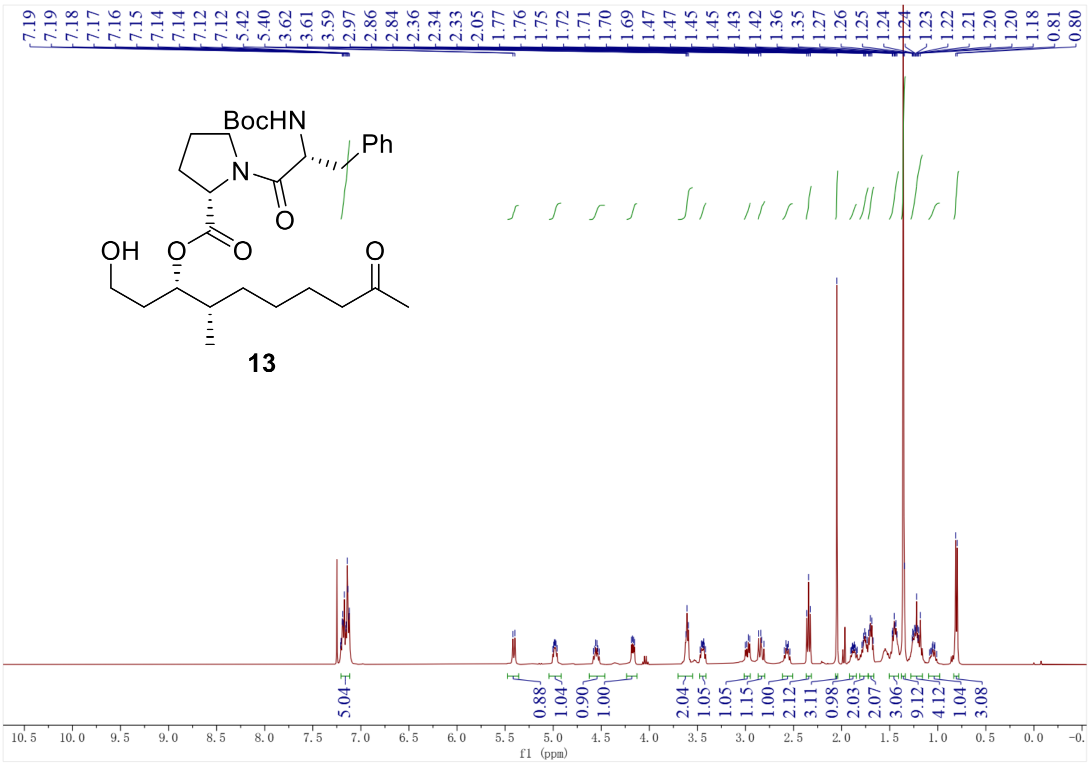

^{13}C NMR Spectra for **13** (101 MHz, CDCl$_3$)

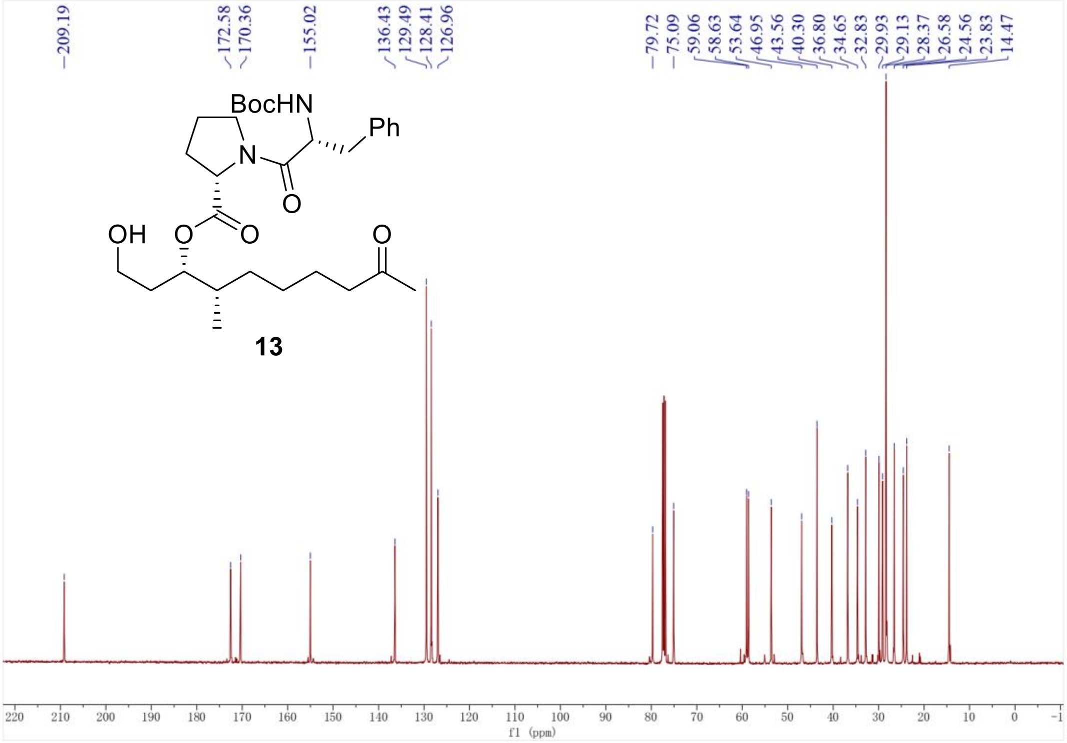

^{1}H NMR Spectra for **15** (400 MHz, $CDCl_3$)

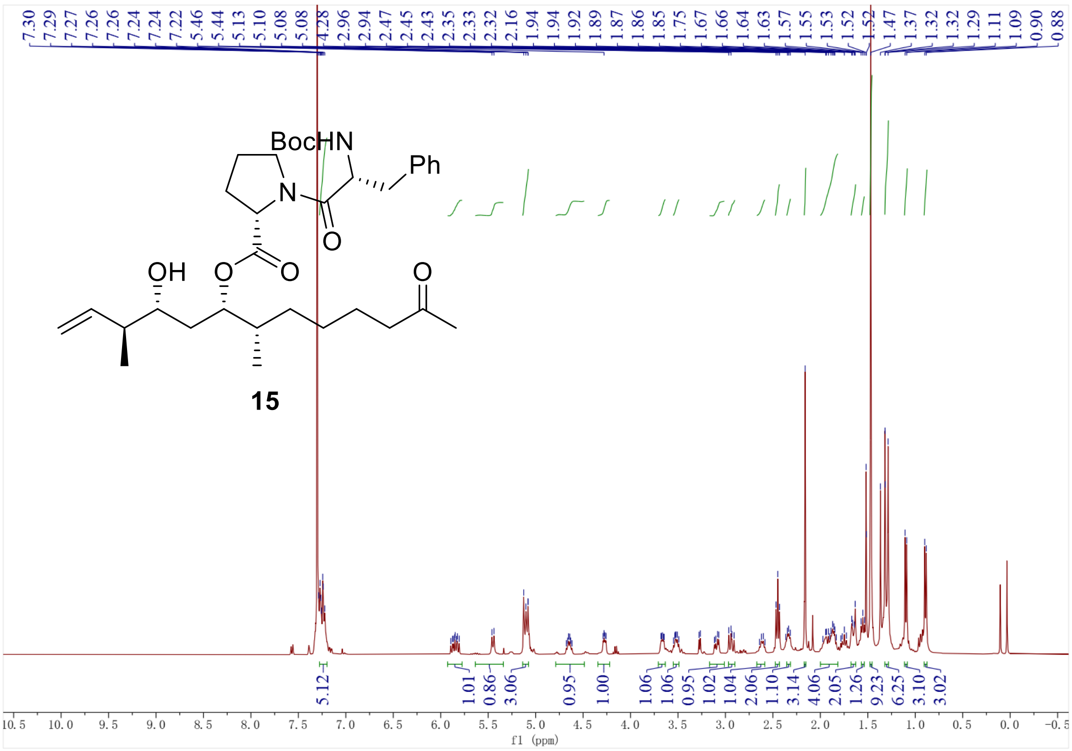

^{13}C NMR Spectra for **15** (101 MHz, $CDCl_3$)

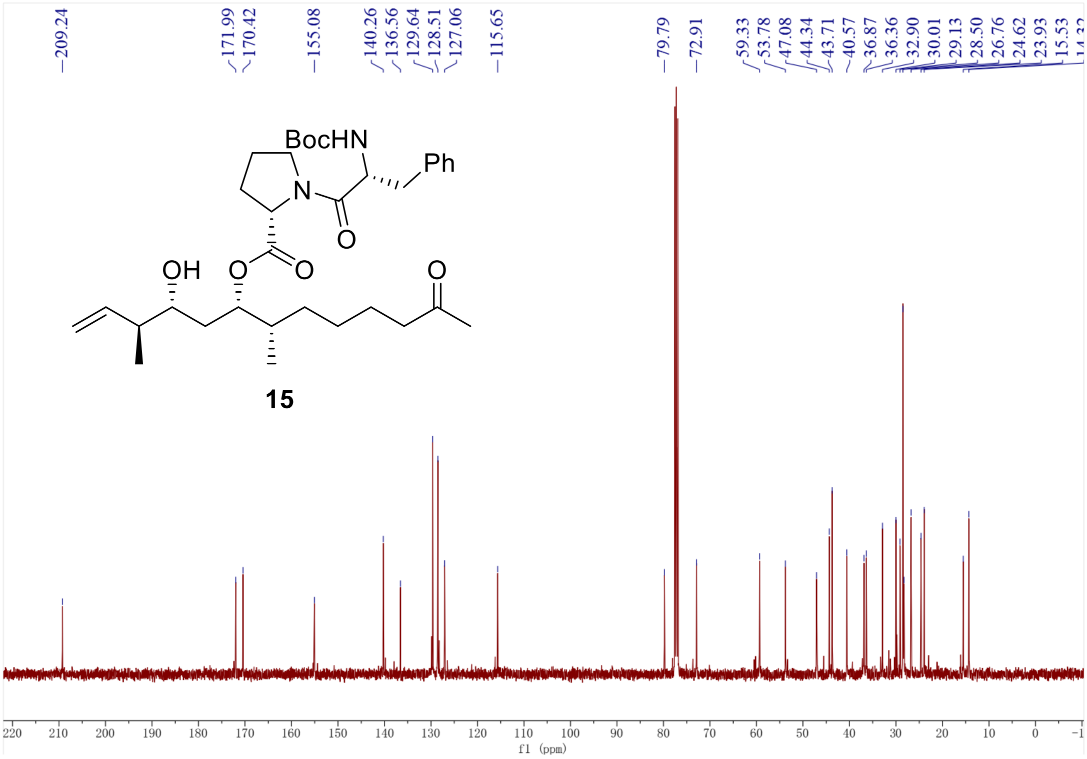

^{1}H NMR Spectra for **16** (400 MHz, $CDCl_3$)

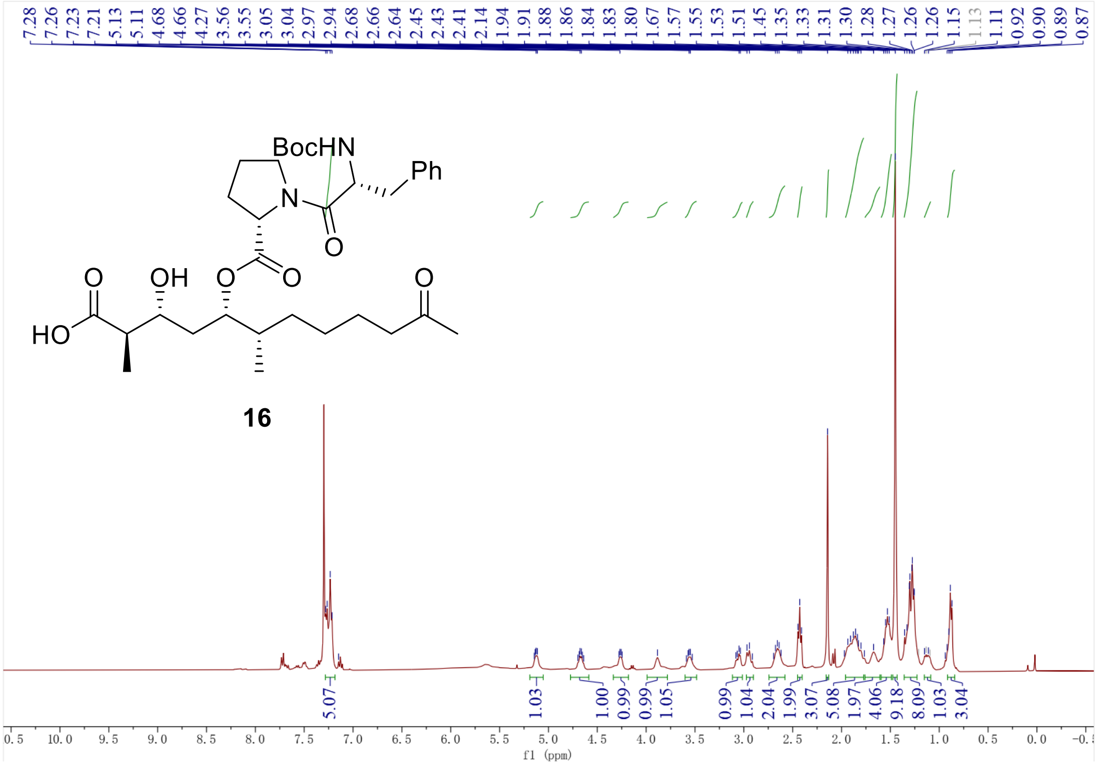

^{13}C NMR Spectra for **16** (101 MHz, $CDCl_3$)

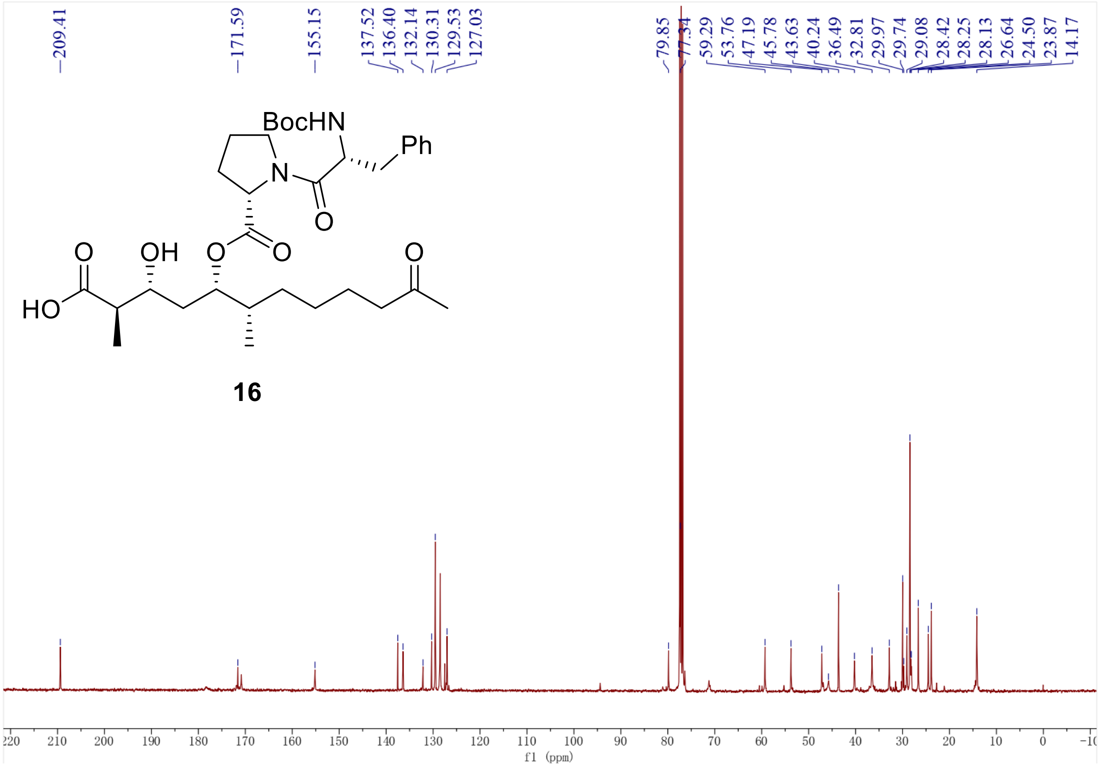

^{1}H NMR Spectra for acremolide B **(2)** (400 MHz, DMSO-d_6)

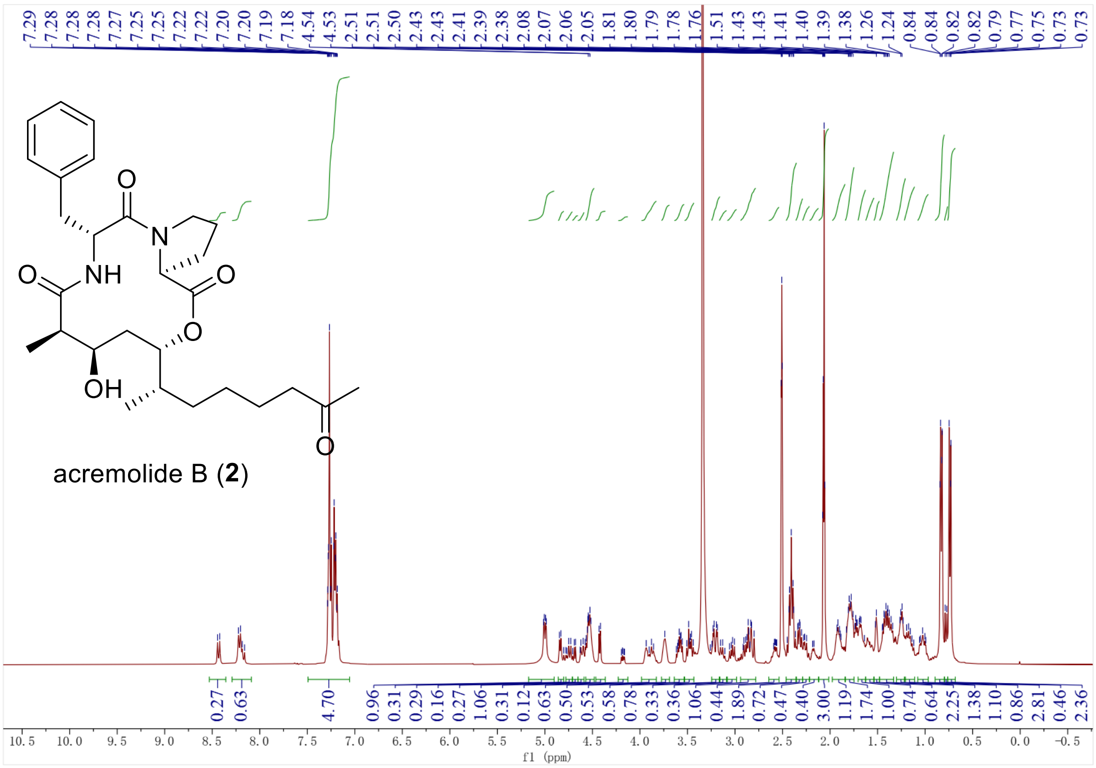

^{13}C NMR Spectra for acremolide B **(2)** (101 MHz, DMSO-d_6)

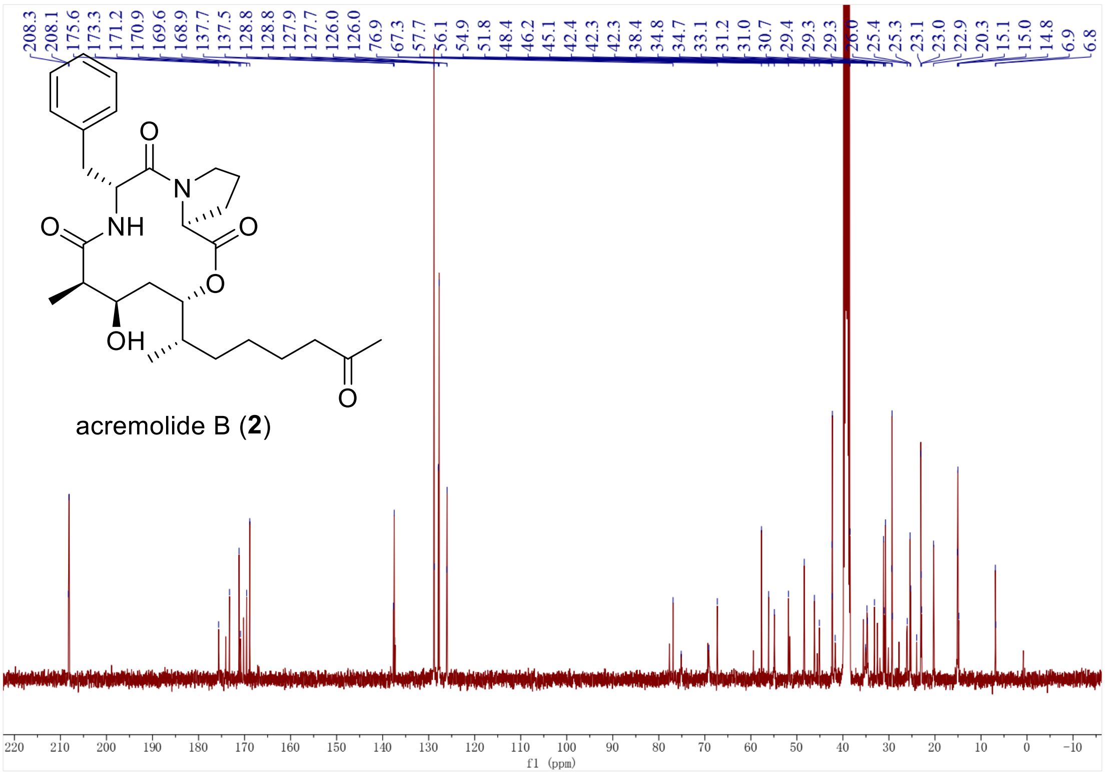

^{1}H NMR Spectra for **18** (400 MHz, $CDCl_3$)

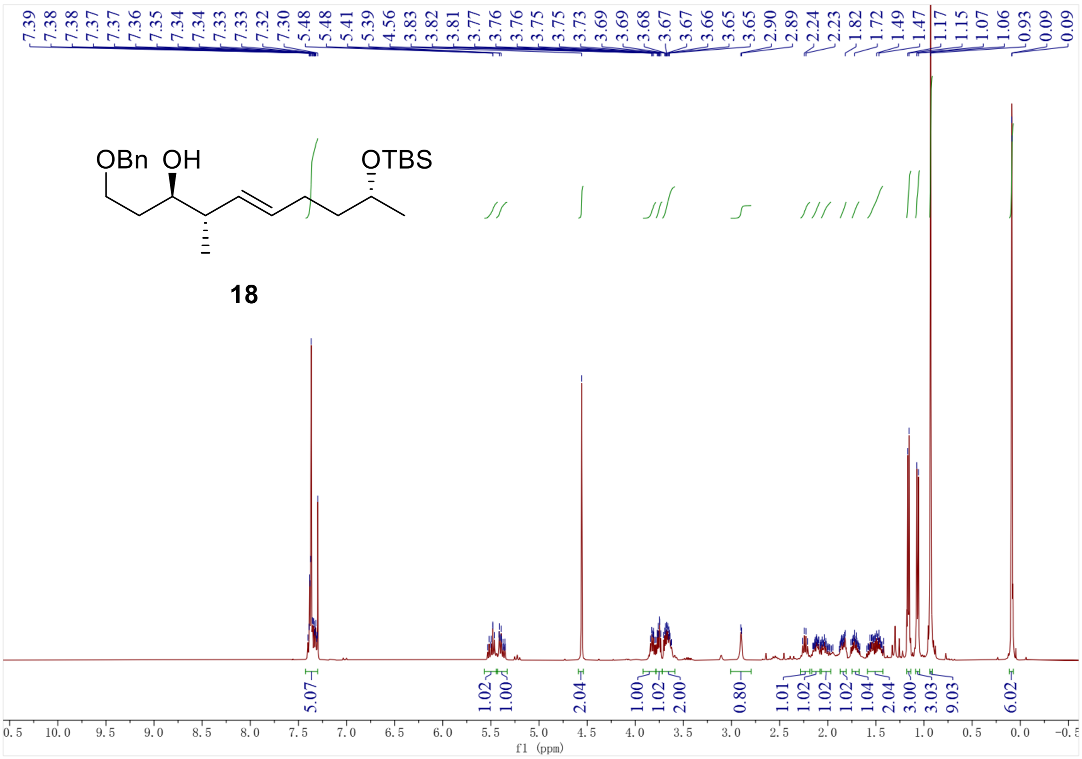

^{13}C NMR Spectra for **18** (101 MHz, $CDCl_3$)

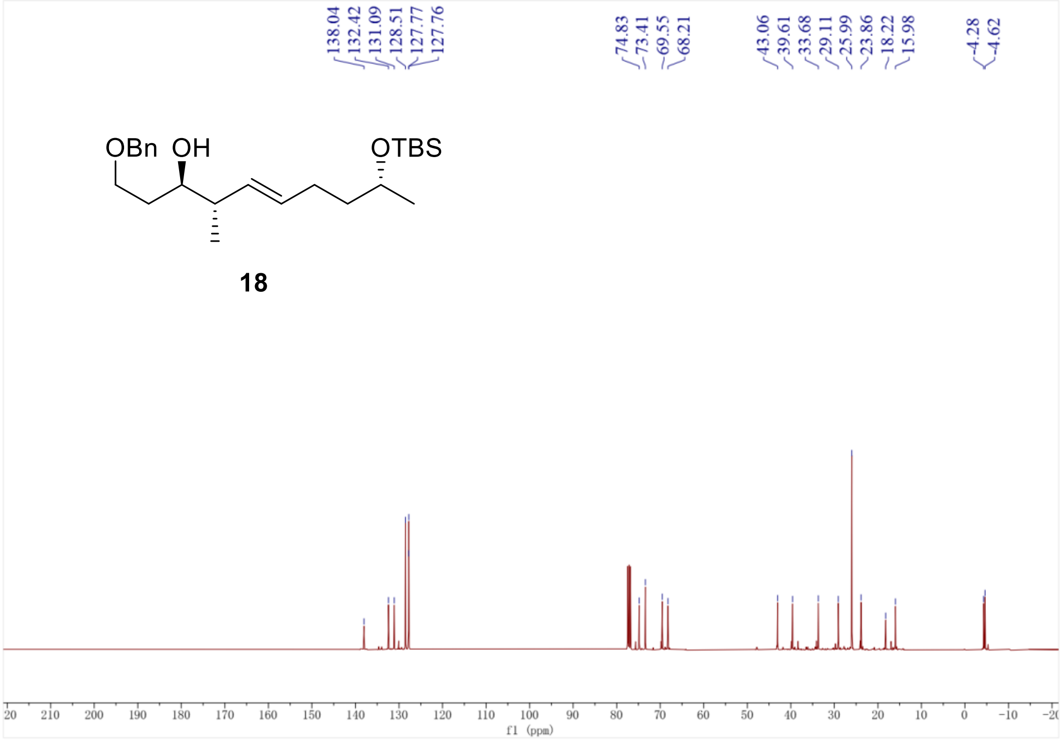

^{1}H NMR Spectra for **19** (400 MHz, $CDCl_3$)

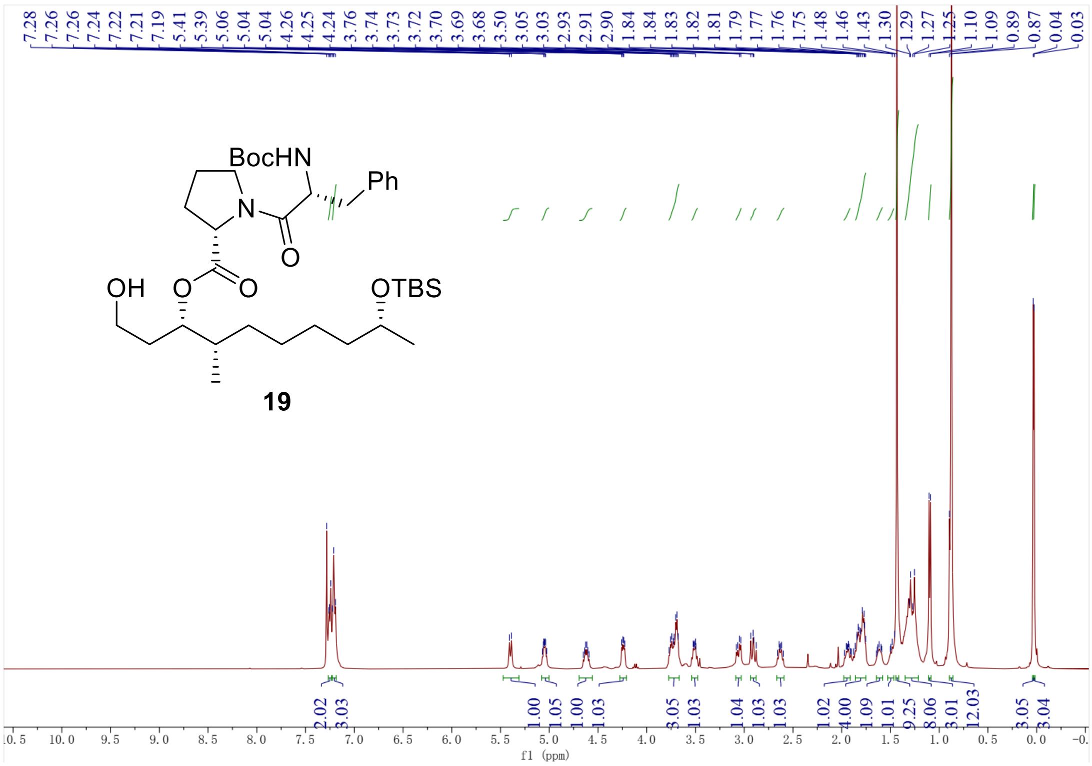

^{13}C NMR Spectra for **19** (101 MHz, $CDCl_3$)

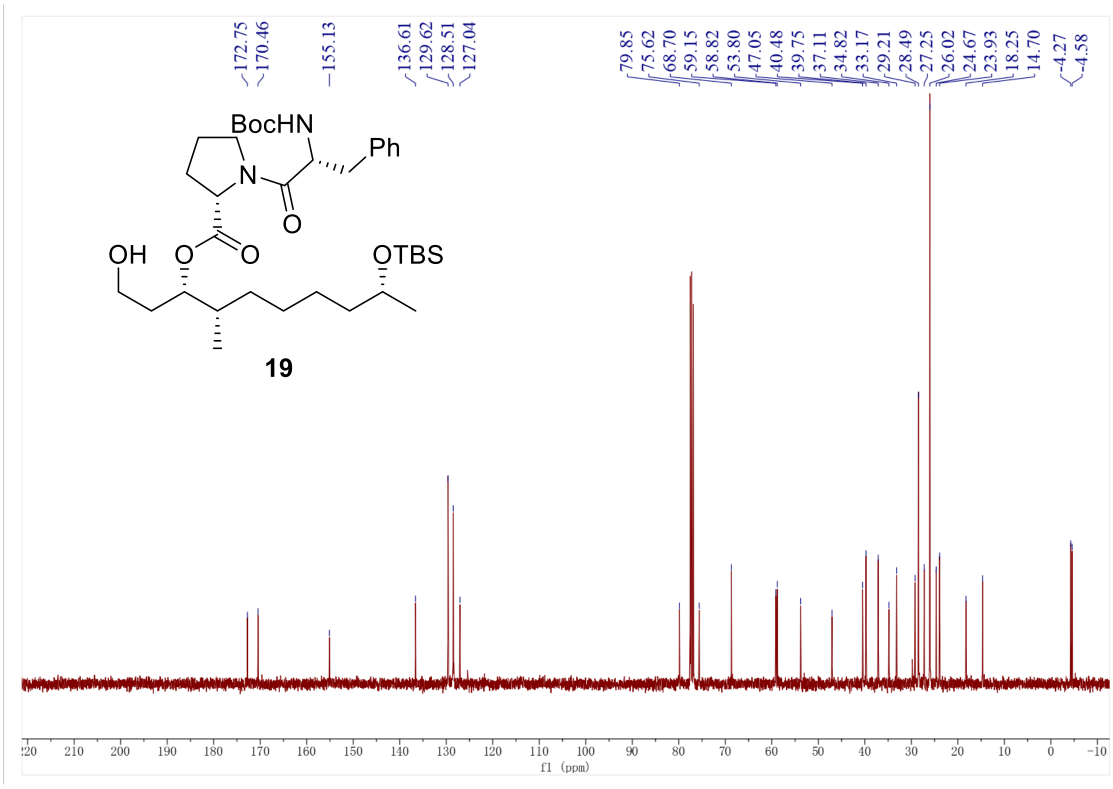

^{1}H NMR Spectra for **20** (400 MHz, $CDCl_3$)

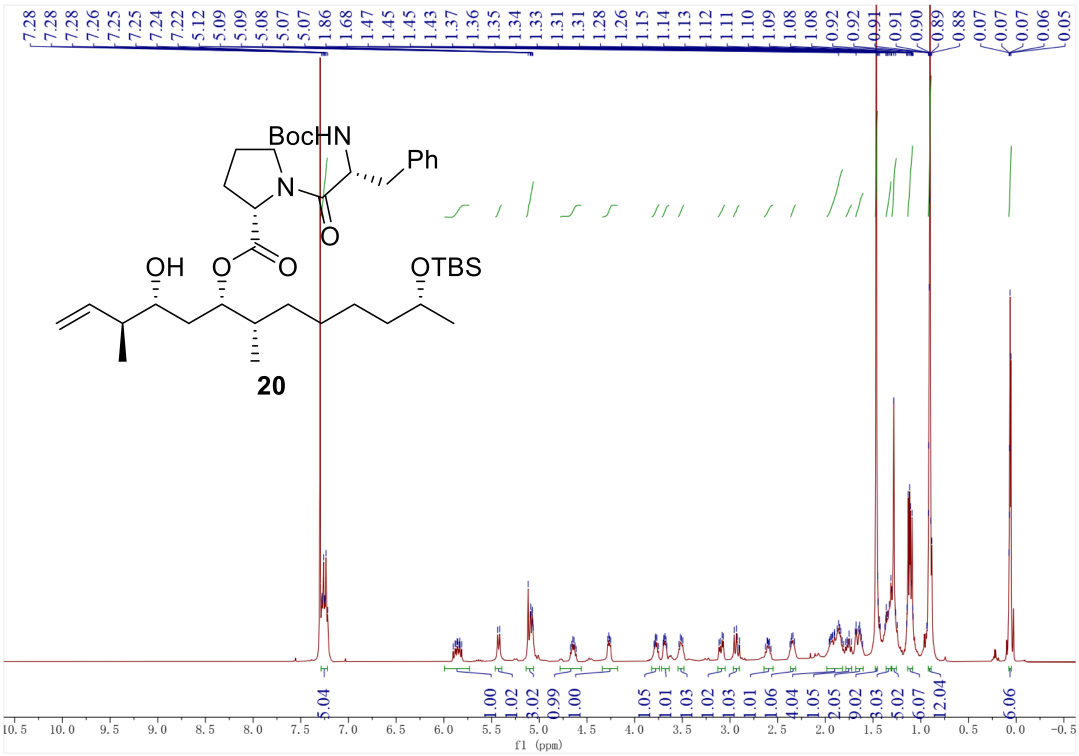

^{13}C NMR Spectra for **20** (101 MHz, $CDCl_3$)

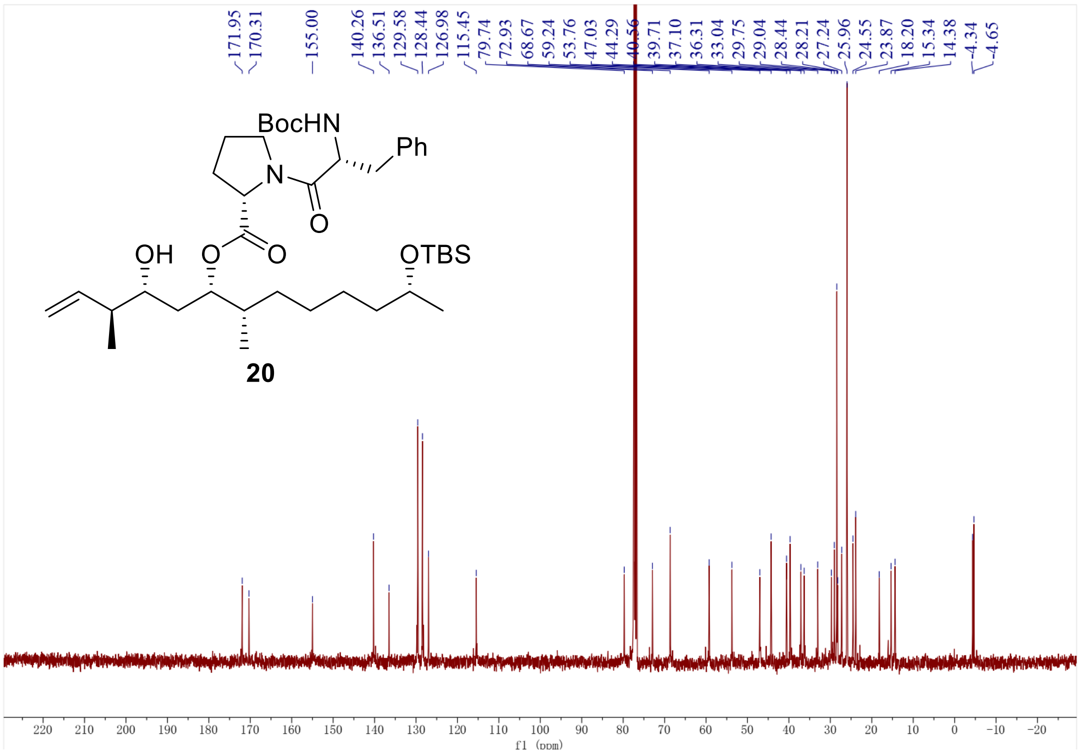

^{1}H NMR Spectra for **21** (400 MHz, $CDCl_3$)

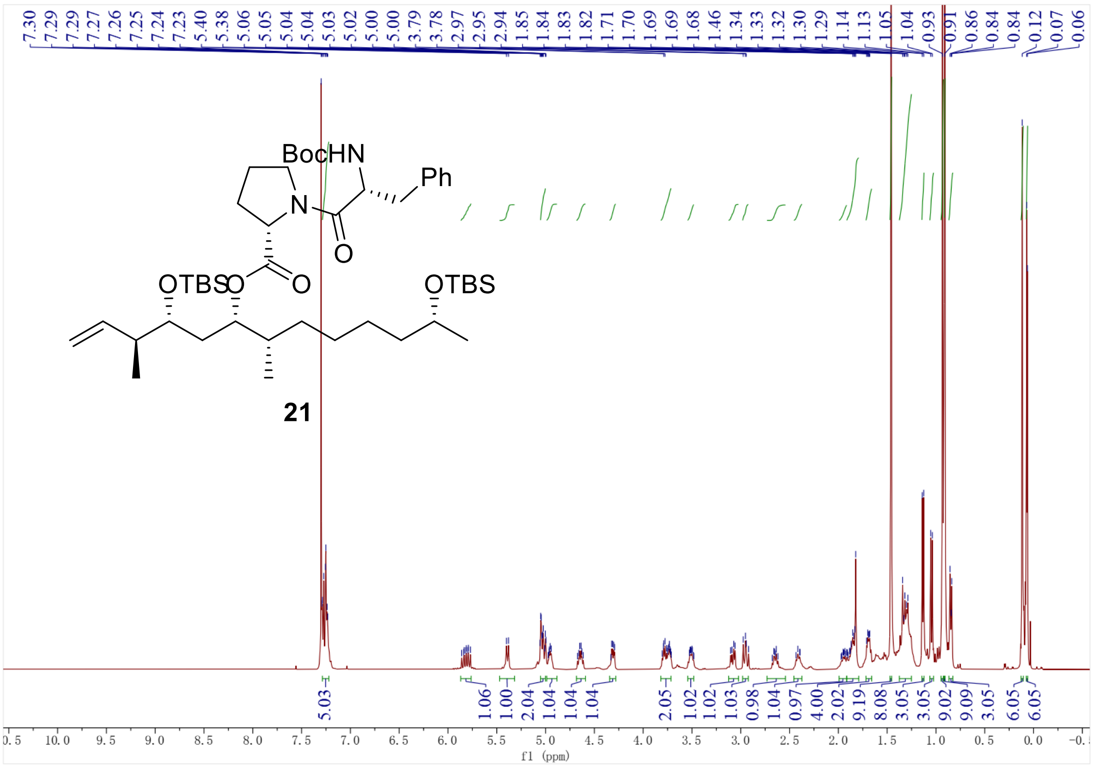

^{13}C NMR Spectra for **21** (101 MHz, $CDCl_3$)

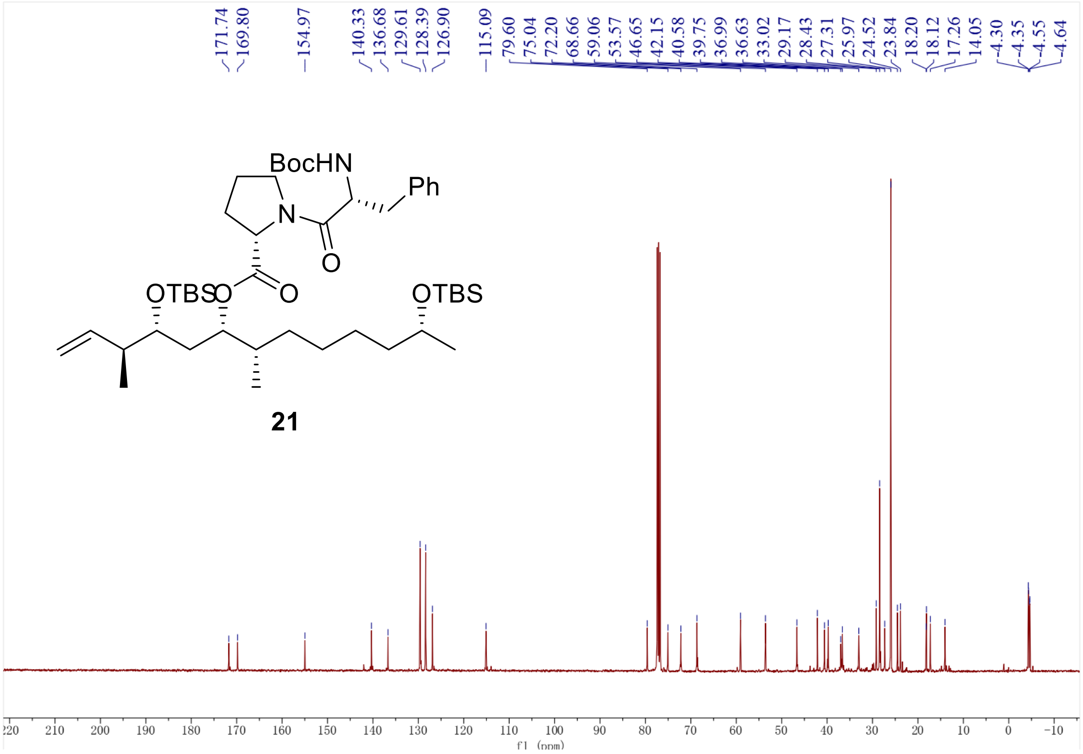

^{1}H NMR Spectra for **22** (400 MHz, $CDCl_3$)

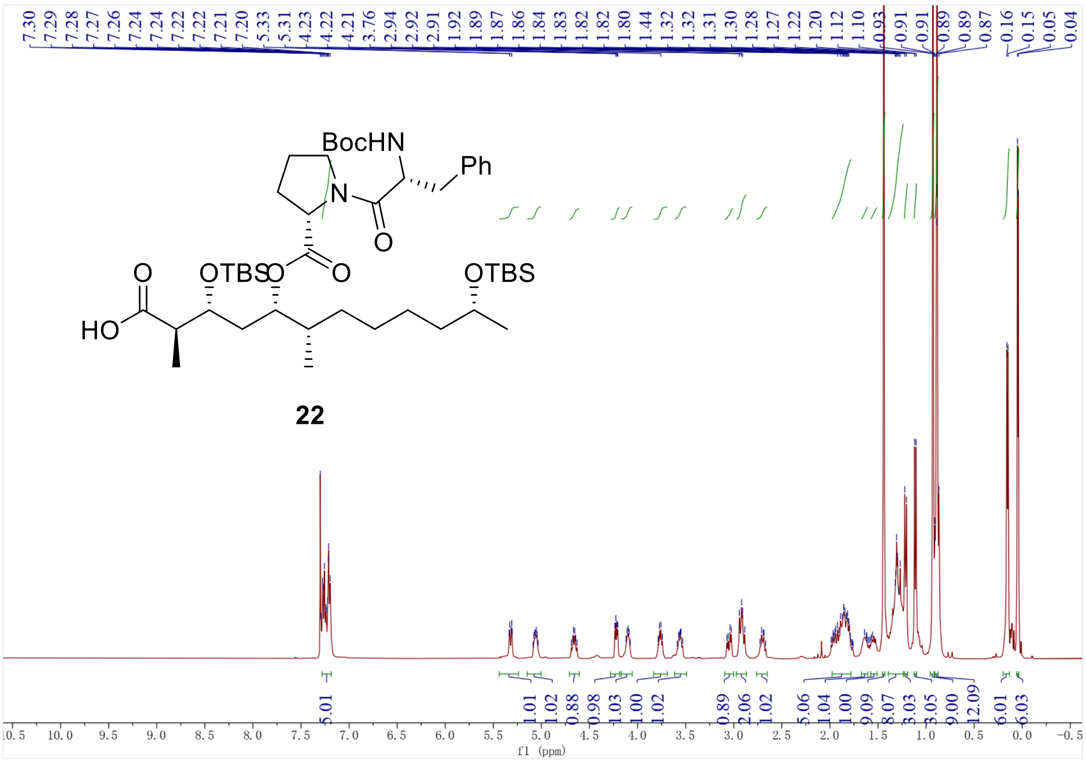

^{13}C NMR Spectra for **22** (101 MHz, $CDCl_3$)

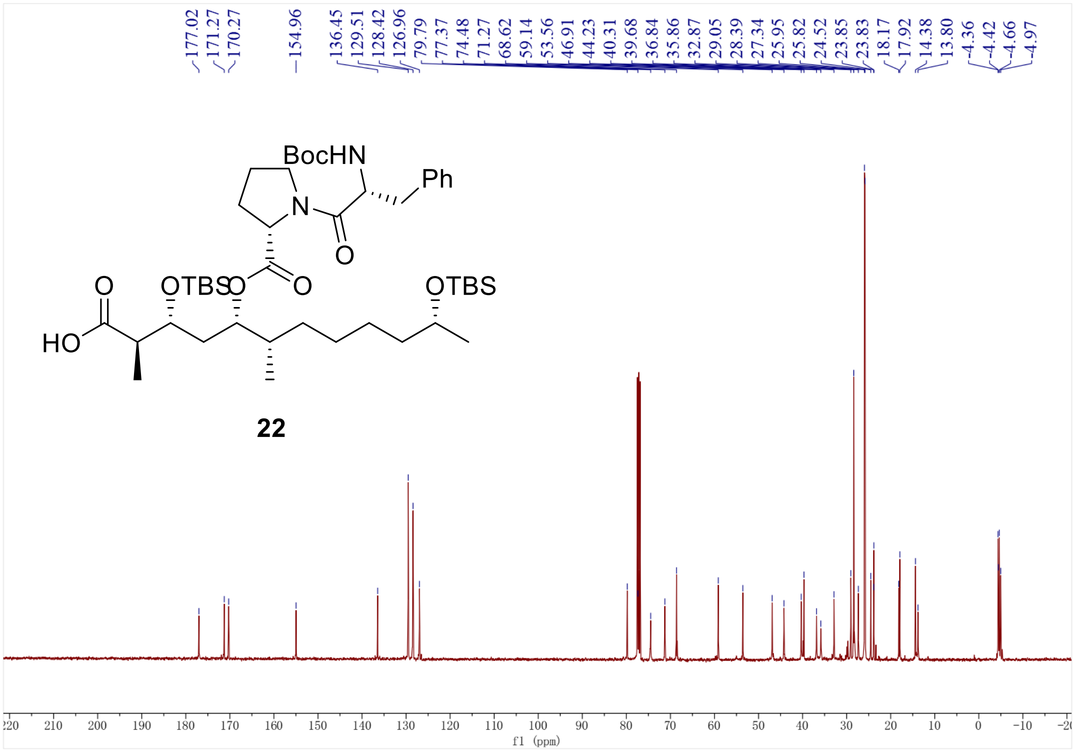

^{1}H NMR Spectra for acremolide A **(1)** (400 MHz, DMSO-d_6)

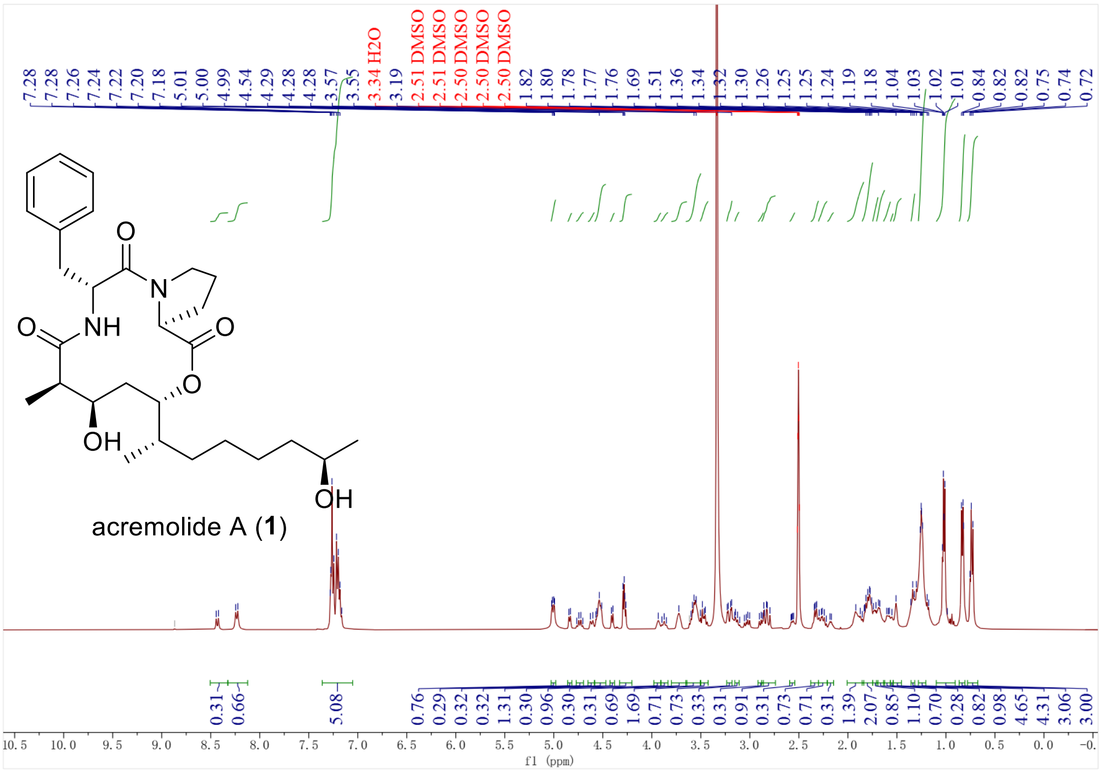

^{13}C NMR Spectra for acremolide A **(1)** (101 MHz, DMSO-d_6)

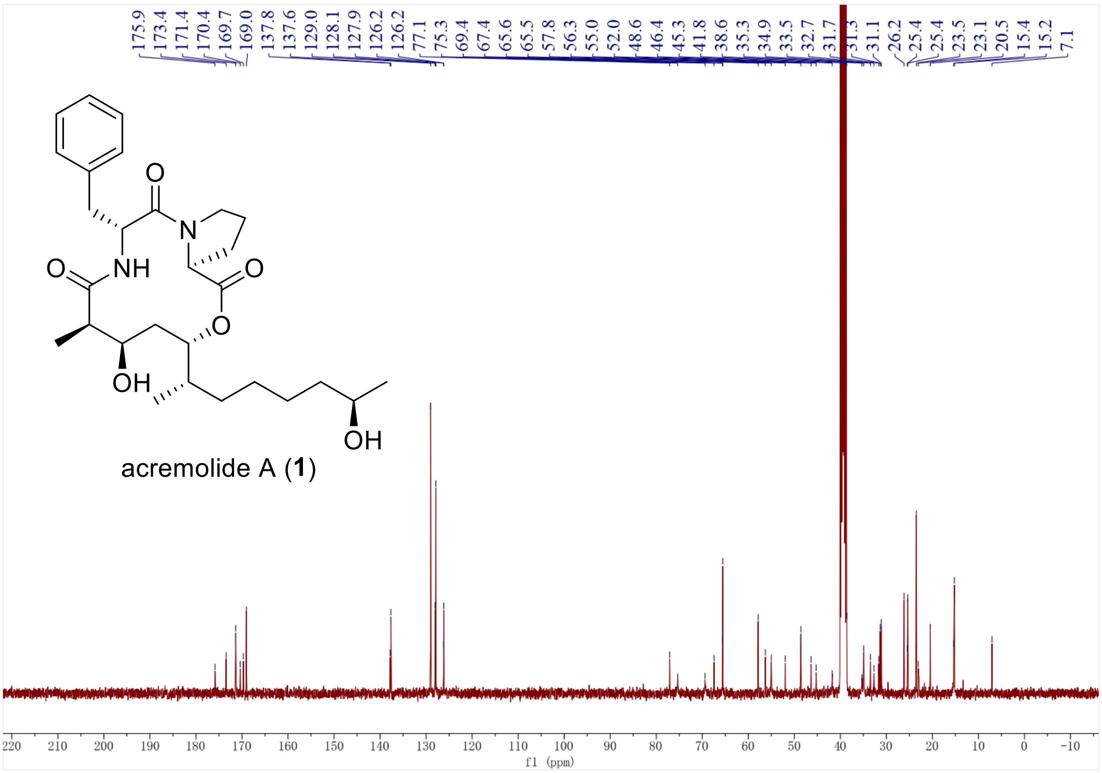

Supplement: Supplementary file 1 [file molecules-29-03599-s001.zip › molecules-3099626-supplementary.pdf]
